# Structural Characterization of Cholestane Rhamnosides from *Ornithogalum saundersiae* Bulbs and Their Cytotoxic Activity against Cultured Tumor Cells

**DOI:** 10.3390/molecules22081243

**Published:** 2017-07-25

**Authors:** Tomoki Iguchi, Minpei Kuroda, Rei Naito, Tomoyuki Watanabe, Yukiko Matsuo, Akihito Yokosuka, Yoshihiro Mimaki

**Affiliations:** School of Pharmacy, Tokyo University of Pharmacy and Life Sciences, 1432-1, Horinouchi, Hachioji, Tokyo 192-0392, Japan; y091015@toyaku.ac.jp (T.I.); 7811imsn@jcom.zaq.ne.jp (R.N.); tmyk.w14@gmail.com (T.W.); matsuoy@toyaku.ac.jp (Y.M.); yokosuka@toyaku.ac.jp (A.Y.); mimakiy@toyaku.ac.jp (Y.M.)

**Keywords:** *Ornithogalum saundersiae*, Asparagaceae, Liliaceae, cholestane rhamnoside, cytotoxicity, HL-60 cells, A549 cells, apoptosis

## Abstract

Previous phytochemical studies of the bulbs of *Ornithogalum saundersiae*, an ornamental perennial plant native to South Africa, resulted in the isolation of 29 new cholestane glycosides, some of which were structurally unique and showed potent cytotoxic activity against cultured tumor cell lines. Therefore, we aimed to perform further phytochemical examinations of methanolic extracts obtained from *Ornithogalum saundersiae* bulbs, isolating 12 new cholestane rhamnosides (**1**–**12**) and seven known compounds (**13**–**19**). The structures of the new compounds (**1**–**12**) were identified via NMR-based structural characterization methods, and through a sequence of chemical transformations followed by spectroscopic and chromatographic analysis. The cytotoxic activity of the isolated compounds (**1**–**19**) and the derivatives (**1a** and **6a**) against HL-60 human promyelocytic leukemia cells and A549 human lung adenocarcinoma cells was evaluated. Compounds **10**–**12**, **16**, and **17** showed cytotoxicity against both HL-60 and A549 cells. Compound **11** showed potent cytotoxicity with an IC_50_ value of 0.16 µM against HL-60 cells and induced apoptotic cell death via a mitochondrion-independent pathway.

## 1. Introduction

*Ornithogalum saundersiae* (Baker), of the Asparagaceae (formerly Liliaceae) family, is an ornamental perennial plant native to South Africa. Our systematic phytochemical examinations of a methanolic extract of *Ornithogalum saundersiae* bulbs resulted in the isolation of 29 new cholestane glycosides including 16,23-epoxy-5β-cholestane glycoside [1], 22-homo-23-norcholestane glycosides [2,3,4,5,6], and polyoxycholestane glycosides [7,8,9,10,11,12]. Some cholestane glycosides, especially those with an aromatic acyl group, showed potent cytotoxic activity against cultured tumor cell lines, including HL-60 human promyelocytic leukemia cells.

**Figure 1:**
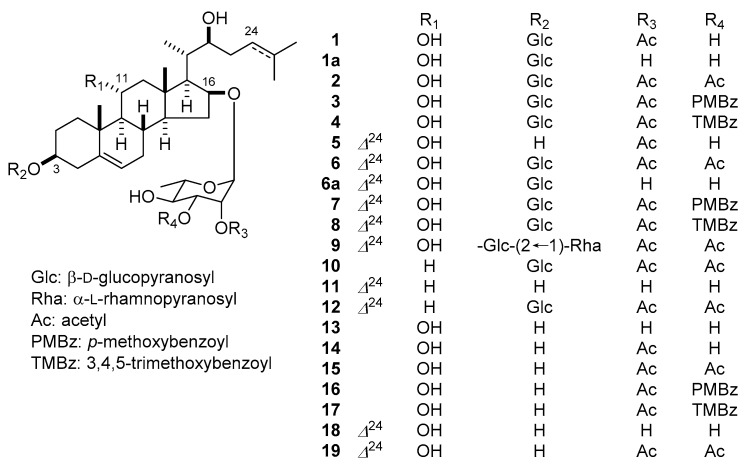
Structures of **1**–**19**, **1a**, and **6a**.

We first isolated 3β,16β,17α-trihydroxycholest-5-en-22-one 16-*O*-(2-*O*-4-methoxybenzoyl-β-d-xylopyranosyl)-(1→3)-(2-*O*-acetyl-α-l-arabinopyranoside), an acylated cholestenol glycoside commonly called OSW-1, from *O. saundersiae* bulbs in 1992 [9]. The cytotoxic activity of OSW-1 against tumor cells was approximately 10–100 times more potent than that of clinically used anticancer agents, with low toxicity against healthy cells [10]. The structural diversity and potent cytotoxic activity of the cholestane glycosides obtained from *O. saundersiae* bulbs prompted us to carry out a further phytochemical examination of the plant. As a result, 12 new (**1**–**12**) and seven known cholestane glycosides (**13**–**19**) (Figure 1) were isolated. The new compounds (**1**–**12**) were identified via NMR-based structural characterization methods, and through a sequence of chemical transformations followed by spectroscopic and chromatographic analysis. The cytotoxic activity of **1**–**19** against HL-60 cells and A549 human lung adenocarcinoma cells, and the apoptosis-inducing properties of **11**, were also investigated.

## 2. Results and Discussion

### 2.1. Isolation and Structure Determination of ***1**–**19***

Fresh bulbs obtained from *O*. *saundersiae* were extracted with hot MeOH. The MeOH extract was passed through a porous polymer polystyrene resin (Diaion HP-20) column. The EtOH-eluted fraction was then subjected to column chromatography (CC) using silica gel and octadecylsilanized (ODS) silica gel, and preparative high-performance liquid chromatography (HPLC), yielding compounds **1**–**19**. The structures of the known compounds were identified as (22*S*)-3β,11α,22-trihydroxycholest-5-en-16β-yl α-l-rhamnopyranoside (**13**) [7], (22*S*)-3β,11α,22-trihydroxycholest-5-en-16β-yl 2-*O*-acetyl-α-l-rhamnopyranoside (**14**) [11], (22*S*)-3β,11α,22-trihydroxycholest-5-en-16β-yl 2,3-di-*O*-acetyl-α-l-rhamnopyranoside (**15**) [7], (22*S*)-3β,11α,22-trihydroxycholest-5-en-16β-yl 2-*O*-acetyl-3-*O*-*p*-methoxybenzoyl-α-L-rhamnopyranoside (**16**) [7], (22*S*)-3β,11α,22-trihydroxycholest-5-en-16β-yl 2-*O*-acetyl-3-*O*-3,4,5-trimethoxybenzoyl-α-l-rhamnopyranoside (**17**) [7], (22*S*)-3β,11α,22-trihydroxycholesta-5,24-dien-16β-yl α-l-rhamnopyranoside (**18**) [7], and (22*S*)-3β,11α,22-trihydroxycholesta-5,24-dien-16β-yl 2,3-di-*O*-acetyl-α-l-rhamnopyranoside (**19**) [7].

Compound **1** was obtained as an amorphous solid, with a specific rotation of −7.1 (MeOH), and the molecular formula C_41_H_68_O_14_ based on the high-resolution electrospray ionization mass spectrometry (HRESIMS, *m*/*z* 807.4518 [M + Na]^+^, calcd. for 807.4507) and ^13^C-NMR spectra. The ^1^H-NMR spectrum of **1** showed signals for five steroidal methyl groups at δ_H_ 1.31 (s, Me-19), 1.23 (d, *J* = 6.8 Hz, Me-21), 1.05 (d, *J* = 6.3 Hz, Me-26), 1.03 (d, *J* = 6.3 Hz, Me-27), 0.99 (s, Me-18); an olefinic proton at δ_H_ 5.44 (br d, *J* = 4.9 Hz, H-6); and two anomeric protons at δ_H_ 5.09 (d, *J* = 7.7 Hz, H-1 of β-d-glucopyranosyl (Glc)) and 5.05 (br s, H-1 of α-l-rhamnopyranosyl (Rha)). Furthermore, the presence of an acetyl group in **1** was revealed via IR (1729 cm^−1^), ^1^H-NMR (δ_H_ 2.03, s, 3H), and ^13^C-NMR (δ_C_ 170.4 C=O and 21.0 Me) spectroscopy (Table 1 and Table 3). Treatment of **1** with 3% NaOMe in MeOH afforded a deacyl derivative (**1a**), and subsequent acid hydrolysis of **1a** with 1 M HCl in dioxane/H_2_O (1:1) yielded (22*S*)-cholest-5-ene-3β,11α,16β,22-tetrol [7] as the aglycone, and d-glucose and l-rhamnose as the carbohydrate moieties. The identification of d-glucose and l-rhamnose, including their absolute configurations, was carried out by direct HPLC analysis of the hydrolysate. These data implied that **1** was a (22*S*)-cholest-5-ene-3β,11α,16β,22-tetrol diglycoside bearing an acetyl group. In the heteronuclear multiple bond correlation (HMBC) spectrum of **1**, long-range correlations were observed between the anomeric proton (H-1) of Glc at δ_H_ 5.09 and C-3 of the aglycone at δ_C_ 78.2, between H-1 of Rha at δ_H_ 5.05 and C-16 of the aglycone at δ_C_ 82.7, and between H-2 of Rha at δ_H_ 5.66 and the carbonyl carbon of the acetyl group at δ_C_ 170.4. All these data indicate that compound **1** structure is (22*S*)-3β-[(β-d-glucopyranosyl)oxy]-11α,22-dihydroxycholest-5-en-16β-yl 2-*O*-acetyl-α-L-rhamnopyranoside.

Compound **2** (C_43_H_70_O_15_) was shown to be closely related to **1** by the ^1^H- and ^13^C-NMR spectra, differing from **1** only by the presence of two acetyl groups moieties (δ_H_ 2.07 and 1.98 (each 3H, s); δ_C_ 170.2 (C=O) and 20.5 (Me), and δ_C_ 170.0 (C=O) and 20.6 (Me)) (Table 1 and Table 3). Alkaline degradation of **2** with 3% NaOMe in MeOH furnished **1a**. In the HMBC spectrum of **2**, correlation peaks were observed between H-2 of Rha at δ_H_ 5.62 and the carbonyl carbon of one acetyl group at δ_C_ 170.0, and between H-3 of Rha at δ_H_ 5.76 and the carbonyl carbon of another acetyl group at δ_C_ 170.2. The structure of **2** was assigned as (22*S*)-3β-[(β-d-glucopyranosyl)oxy]-11α,22-dihydroxycholest-5-en-16β-yl 2,3-di-*O*-acetyl-α-l-rhamnopyranoside.

Compound **3** (C_49_H_74_O_16_) and **4** (C_51_H_78_O_18_) were suggested to be essentially analogous to **2** based on the ^1^H- and ^13^C-NMR spectra; however, the signals for one acetyl group attached to the rhamnosyl group were not present. Instead, the signals assignable to a *p*-methoxybenzoyl group and a 3,4,5-trimethoxybenzoyl group were detectable in the ^1^H- and ^13^C-NMR spectra of **3** and **4**, respectively. Upon alkaline methanolysis of **3** and **4** with 3% NaOMe in MeOH, methyl *p*-methoxybenzoate and **1a** were obtained from **3**, while methyl 3,4,5-trimethoxybenzoate and **1a** were obtained from **4**. HMBC analysis of **3** revealed long-range correlations between H-2 of Rha at δ_H_ 5.78 (dd, *J* = 3.0, 1.9 Hz) and the carbonyl carbon of the acetyl group at δ_C_ 169.9, and between H-3 of Rha at δ_H_ 6.05 (dd, *J* = 9.4, 3.0 Hz) and the carbonyl carbon of the *p*-methoxybenzoyl group at δ_C_ 165.8. Similarly, correlation peaks were observed between H-2 of Rha at δ_H_ 5.81 (dd, *J* = 3.3, 1.7 Hz) and the carbonyl carbon of the acetyl group at δ_C_ 170.0, and between H-3 of Rha at δ_H_ 6.08 (dd, *J* = 9.6, 3.3 Hz) and the carbonyl carbon of the 3,4,5-trimethoxybenzoyl group at δ_C_ 165.9 in that of **4**. Thus, the structures of **3** and **4** were elucidated as (22*S*)-3β-[(β-d-glucopyranosyl)oxy]-11α,22-dihydroxycholest-5-en-16β-yl 2-*O*-acetyl-3-*O*-*p*-methoxybenzoyl-α-L-rhamnopyranoside and (22*S*)-3β-[(β-d-glucopyranosyl)oxy]-11α,22-dihydroxycholest-5-en-16β-yl 2-*O*-acetyl-3-*O*-(3,4,5-trimethoxybenzoyl)-α-l-rhamnopyranoside, respectively.

Compound **5** (C_35_H_56_O_9_) was obtained as an amorphous powder. The ^1^H-NMR spectrum of **5** displayed signals arising from two tertiary methyl groups at δ_H_ 1.41 and 1.00 (each s, Me-19, Me-18); a secondary methyl group at δ_H_ 1.26 (d, *J* = 6.8 Hz, Me-21); two methyl groups on a double bond at δ_H_ 1.78 (Me-27) and 1.73 (Me-26); two olefinic protons at δ_H_ 5.82 (br t, *J* = 7.2 Hz, H-24) and 5.65 (br d, *J* = 4.7 Hz, H-6); an anomeric proton at δ_H_ 5.03 (br s, H-1 of Rha); and the methyl group of an acetyl moiety at δ_H_ 2.03 (s). These spectral features of **5** showed similarity to those of **18**, and alkaline degradation of **5** with 3% NaOMe in MeOH yielded **18**. A long-range correlation was observed between the H-2 of Rha at δ_H_ 5.65 (br d, *J* = 2.2 Hz) and the carbonyl carbon of the acetyl group at δ_C_ 170.6 in the HMBC spectrum of **5**, providing evidence that the acetyl group was located at C-2 of the α-l-rhamnopyranosyl moiety. The structure of **5** was defined as (22*S*)-3β,11α,22-trihydroxycholesta-5,24-dien-16β-yl 2-*O*-acetyl-α-l-rhamnopyranoside.

Compound **6** (C_43_H_68_O_15_), for which ^1^H-NMR analysis showed two anomeric proton signals at δ_H_ 5.07 (d, *J* = 7.7 Hz, H-1 of Glc) and 5.03 (br s, H-1 of Rha), was assumed to be a bisdesmosidic cholestane glycoside closely related to **2**. The two compounds differed only in the signals of the methyl groups assignable to Me-26 and Me-27. The two three-proton doublets for Me-26 and Me-27 observed in the ^1^H-NMR spectrum of **2** were displaced by two three-proton singlets at δ_H_ 1.77 and 1.73 in that of **6**, suggesting that **6** was the C-24/C-25 dehydro derivative of **2**. This was confirmed by alkaline methanolysis of **6**, followed by enzymatic hydrolysis using β-d-glucosidase, to yield **18** and d-glucose. The linkage position of the glucosyl moiety of **6** was ascertained by an HMBC correlation between H-1 of Glc and C-3 of the aglycone at δ_C_ 78.1. The structure of **6** was elucidated as (22*S*)-3β-[(β-d-glucopyranosyl)oxy]-11α,22-dihydroxycholesta-5,24-dien-16β-yl 2,3-di-*O*-acetyl-α-l-rhamnopyranoside.

Compound **7** (C_49_H_72_O_16_) and **8** (C_51_H_76_O_18_) were suggested to be the corresponding C-24/C-25 dehydro derivatives of **3** and **4**, respectively, based on their spectral properties (Table 2 and Table 3). This assumption was subsequently verified by chemical and spectroscopic analysis. When **7** and **8** were treated with 3% NaOMe, **7** yielded methyl *p*-methoxybenzoate and **6a**, while **8** furnished methyl 3,4,5-trimethoxybenzoate and **6a**. In the HMBC spectra of **7**, long-range correlations were observed between H-2 of Rha at δ_H_ 5.78 (dd, *J* = 2.7, 1.4 Hz) and the carbonyl carbon of the acetyl group at δ_C_ 170.0, and between H-3 of Rha δ_H_ 6.08 (dd, *J* = 9.7, 2.7 Hz) and the carbonyl carbon of the *p*-methoxybenzoyl group at δ_C_ 165.9. Similarly in **8**, correlation peaks were observed between H-2 of Rha at δ_H_ 5.81 (dd, *J* = 3.1, 1.6 Hz) and the carbonyl carbon of the acetyl group at δ_C_ 170.1, and between H-3 of Rha at δ_H_ 6.11 (dd, *J* = 9.6, 3.1 Hz) and the carbonyl carbon of the 3,4,5-trimethoxybenzoyl group at δ_C_ 166.0. The structures of **7** and **8** were assigned as (22*S*)-3β-[(β-d-glucopyranosyl)oxy]-11α,22-dihydroxycholesta-5,24-dien-16β-yl 2-*O*-acetyl-3-*O*-*p*-methoxybenzoyl-α-l-rhamnopyranoside and (22*S*)-3β-[(β-d-glucopyranosyl)oxy]-11α,22-dihydroxycholesta-5,24-dien-16β-yl 2-*O*-acetyl-3-*O*-(3,4,5-trimethoxybenzoyl)-α-l-rhamnopyranoside, respectively.

Compound **9** was obtained as an amorphous solid and shown to have the molecular formula C_49_H_78_O_19_ based on HRESIMS (*m*/*z*: 993.5050 [M + Na]^+^, calcd. 993.5035) and ^13^C-NMR spectral data. The deduced molecular formula of **9** was higher than that of **6** by C_6_H_10_O_4_, and the ^1^H-NMR spectrum showed signals for three anomeric protons at δ_H_ 6.39 and 5.07 (each, br s), and 5.07 (d, *J* = 7.3 Hz), and the methyl groups of two 6-deoxy sugars at δ_H_ 1.75 (3H × 2, d, *J* = 6.0 Hz). Acid hydrolysis of **9** yielded l-rhamnose and d-glucose. When the ^13^C-NMR spectrum of **9** was compared with that of **6** (Table 3), a set of signals corresponding to one more terminal α-l-rhamnopyranosyl unit (Rha’) (δ_C_ 102.1 (CH), 72.6 (CH), 72.9 (CH), 74.2 (CH), 69.5 (CH), and 18.7 (Me)) were observed. The resonances attributable to C-2 of the inner Glc unit and its neighboring carbons varied, while all the other signals remained almost unaffected. In the HMBC spectrum of **9**, long-range correlations were observed between H-1 of Rha at δ_H_ 6.39 (br s) and C-2 of Glc at δ_C_ 77.9, and between H-1 of Glc at δ_H_ 5.07 (*J* = 7.3 Hz) and C-3 of the aglycone at δ_C_ 78.2. Thus, the structure of **9** was determined as (22*S*)-3β-[(α-l-rhamnopyranosyl-(1→2)-β-d-glucopyranosyl)oxy]-11α,22-dihydroxycholesta-5,24-dien-16β-yl 2,3-di-*O*-acetyl-α-l-rhamnopyranoside.

Compound **10** (C_43_H_70_O_14_) was suggested to be a bisdesmosidic cholestane glycoside closely related to **2**, both possessing acetyl groups at C-2 and C-3 of the α-l-rhamnopyranosyl moiety attached to the aglycone’s C-16 hydroxy group. However, the molecular formula of **10** contained one oxygen atom less than **2**. When the ^13^C-NMR spectrum of **10** was compared with that of **2**, the hydroxymethine carbon signal observed at δ_C_ 67.8 (C-11) in the spectrum of**2** was displaced by a methylene carbon signal at δ_C_ 21.1 in that of **10**, indicating that **10** was the C-11 dehydroxy derivative of **2**. Alkaline methanolysis of 10 yielded the structure (22*S*)-3β-[(β-d-glucopyranosyl)oxy]-22-hydroxycholest-5-en-16β-yl α-l-rhamnopyranoside (**10a**), which we have previously isolated from *Galtonia candicans* [13]. The structure of **10** was assigned as (22*S*)-3β-[(β-d-glucopyranosyl)oxy]-22-hydroxycholest-5-en-16β-yl 2,3-di-*O*-acetyl-α-l-rhamnopyranoside.

Compound **11** (C_33_H_54_O_8_) was a cholestane rhamnoside with similar spectral properties to those of **18**. However, the molecular formula of **11** lacked one oxygen atom in comparison to **18**, and the ^13^C signals assignable to C-11 differed between the two compounds. The hydroxymethine carbone signal observed at δ_C_ 68.1 (C-11) in the ^13^C-NMR spectrum of **18** was displaced by a methylene carbon signal at δ_C_ 21.1 in that of **11**, allowing **11**’s structure to be determined as (22*S*)-3β,22-dihydroxycholesta-5,24-dien-16β-yl α-l-rhamnopyranoside.

Compound **12** had the molecular formula C_43_H_68_O_14_, possessing two fewer hydrogen atoms than **10**. The two three-proton doublets for Me-26 and Me-27 observed in the ^1^H-NMR spectrum of **10** were displaced by two three-proton singlets at δ_H_ 1.79 and 1.75 in that of **12**, suggesting that **12** was the C-24/C-25 dehydro derivative of **10**. Catalytic hydrogenation of **12** over Pd-C in an H_2_ atmosphere yielded **10**. Thus, the structure of **12** was formulated as (22*S*)-3β-[(β-d-glucopyranosyl)oxy]-22-hydroxycholesta-5,24-dien-16β-yl 2,3-di-*O*-acetyl-α-l-rhamnopyranoside.

### 2.2. Cytotoxic Activity of ***1**–**19***, ***1a***, and ***6a***

The cytotoxic activity of the isolated compounds (**1**–**19**) and the deacetyl derivatives (**1a** and **6a**) against HL-60 cells was evaluated using a modified 3-(4,5-dimethylthiazol-2-yl)-2,5-diphenyl-2*H*-tetrazolium bromide (MTT) assay (Table 4). Compounds **10**–**12**, **16**, and **17** showed potent cytotoxic activity against HL-60 cells, with IC_50_ values ranging from 0.05 to 0.16 μM; etoposide and cisplatin were used as positive controls and gave IC_50_ values of 0.23 and 1.52 µM, respectively. The hydrophilic substituents of the hydroxy group at C-11 and/or the glucosyl group at C-3 in the steroidal frame reduced the cytotoxicity of these compounds. The cholestane rhamnoside lacking these substituents (**11**) showed considerable cytotoxicity to HL-60 cells, with an IC_50_ value of 0.16 µM. On the contrary, acylation of the C-2 hydroxy group of the rhamnosyl moiety with acetic acid, and the C-3 hydroxy group with an acetic acid or a benzoic acid derivative, enhanced the cytotoxicity. Although **10** and **12** had a glucosyl moiety at the C-3 hydroxy group of the aglycone, and **16** and **17** had a hydroxy group at C-11 of the aglycone, they simultaneously possessed acyl groups at C-2 and C-3 of the rhamnosyl moiety and exhibited more potent cytotoxic activity against HL-60 cells than the positive controls. Compounds **10**–**12**, **16**, and **17** also showed significant cytotoxic activity against A549 cells (Table 5).

### 2.3. Apoptosis-Inducing Properties of ***11*** in HL-60 Cells

**Figure 2:**
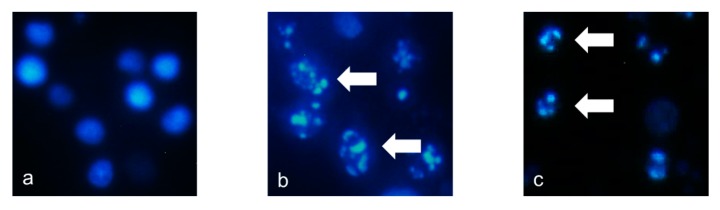
Representative fields showing the morphology of HL-60 cells stained with DAPI after the following treatments for 24 h (magnification, 200×): (**a**) control (**b**) 15 µM of etoposide, or (**c**) 10 µM of **11**. The arrows indicate fragmented and condensed nuclear chromatin.

**Figure 3:**
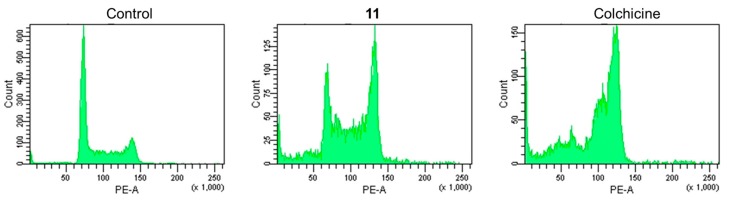
Effects of **11** or colchicine on cell cycle progression in HL-60 cells. Cell cycle distribution in HL-60 cells after treatment with 10 µM of **11** or 1 µM of colchicine for 20 h.

**Figure 4:**
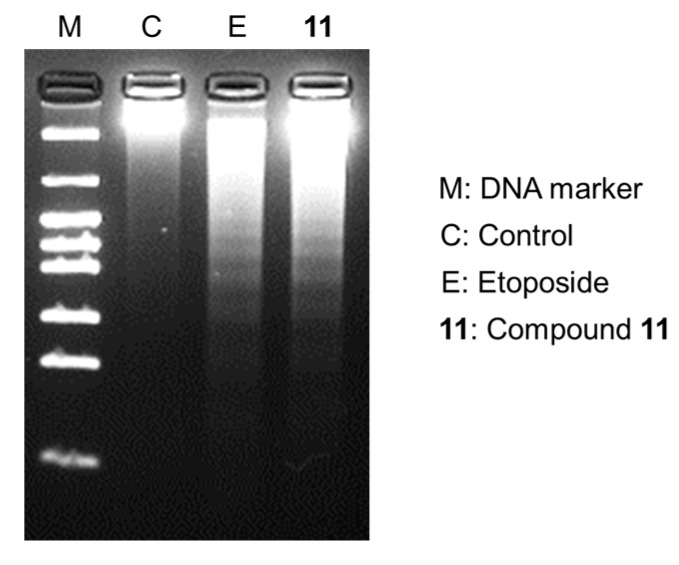
Induction of DNA fragmentation by **11** in HL-60 cells. HL-60 cells were incubated at 37 °C for 20 h with 10 µM of **11** or 15 µM of etoposide (E). DNA was then extracted and subjected to agarose gel electrophoresis.

**Figure 5:**
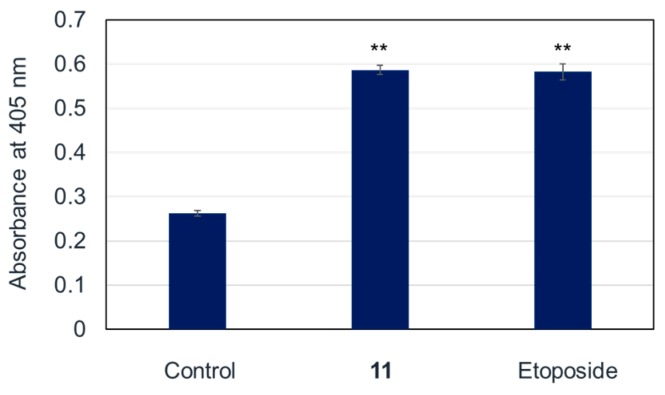
Caspase-3 activity in the lysates of cells treated with **11** or etoposide. HL-60 cells were incubated at 37 °C for 24 h with 10 µM of **11** or 15 µM of etoposide. The data are presented as the mean ± S.E.M. of three experiments. ** *p* < 0.01 vs. the control group readings.

As described above, the new cholestane rhamnoside (**11**) showed potent cytotoxicity against HL-60 cells. Therefore, the apoptosis-inducing properties of **11** in HL-60 cells were evaluated. After HL-60 cells were exposed to **11** at a concentration of 10 µM for 24 h, the cells were stained with 4′,6-diamidino-2-phenylindole dihydrochloride (DAPI) and observed via fluorescence microscopy. The cells exhibited nuclear chromatin condensation and nuclear disassembly as shown in Figure 2, which are the representative morphological features of apoptotic cells. To obtain further evidence for the apoptosis-inducing activity of **11**, cell cycle distribution, DNA fragmentation, and caspase-3 activity were evaluated in **11**-treated HL-60 cells. When HL-60 cells were cultured with **11** at 10 µM for 20 h, the sub-G1 and G2/M phase populations, of which the vehicle control were 3.3 ± 0.20% and 22.4 ± 0.10%, increased to 12.3 ± 0.70% and 32.2 ± 2.44%, respectively (Figure 3). This showed that **11** arrested HL-60 cell proliferation in the G2/M phase and induced apoptotic cell death. Agarose gel electrophoresis of the DNA fraction of HL-60 cells treated with **11** at 10 µM for 20 h displayed an apoptotic DNA ladder pattern, as shown in Figure 4. Furthermore, caspase-3, a key enzyme in the execution phase of the apoptotic pathway, was markedly activated in HL-60 cells treated with **11** at 10 µM for 20 h (Figure 5). These results indicated that HL-60 cell death was partially mediated by **11** via the induction of apoptosis.

### 2.4. The Pathway of Apoptosis Induced by ***11***

**Figure 6:**
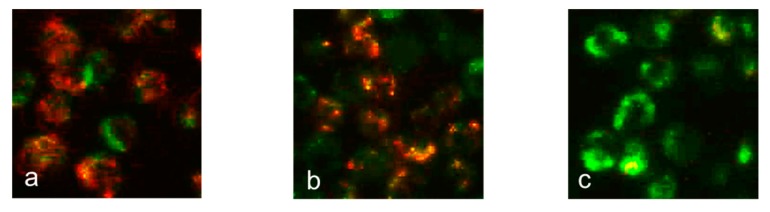
Effects of **11** or cisplatin on mitochondrial membrane potential in HL-60 cells. HL-60 cells were stained with MitoCapture^TM^ reagent after the following treatments for 6 h (magnification, 200×): (**a**) control; (**b**) 10 µM of **11**; and (**c**) 33 µM of cisplatin. Red fluorescence indicates accumulation of MitoCapture^TM^ dye in the mitochondria due to normal mitochondrial membrane potential. Green fluorescence indicates cytosolic localization of MitoCapture^TM^ dye in its monomeric form due to altered mitochondrial membrane potential.

**Figure 7:**
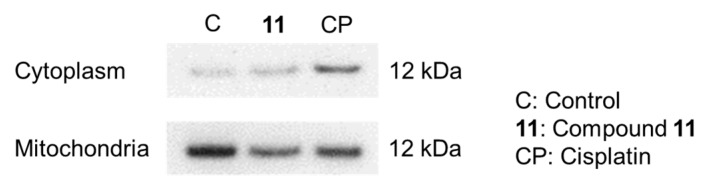
Effects of treatment with 10 µM of **11** or 33 µM of etoposide for 6 h on protein expression of cytochrome *c* in mitochondrial and cytosolic fractions of HL-60 cells.

Apoptotic HL-60 cell death, induced by **11**, was examined to ascertain whether this occurred via a mitochondria-dependent or -independent pathway. When stained with MitoCapture^TM^ dye, apoptotic cells emit green fluorescence, whereas non-apoptotic cells with healthy mitochondria emit red fluorescence. As shown in Figure 6, HL-60 cells treated with cisplatin at 33 µM for 6 h showed a decrease in red fluorescence intensity compared to that of control cells. However, treatment of HL-60 cells with **11** at 10 µM for 6 h did not decrease the intensity of red fluorescence, indicating that **11** caused no disruption of the mitochondrial membrane potential in HL-60 cells (Figure 6). An increase in mitochondrial membrane permeability and a decrease in mitochondrial membrane potential resulted in the rapid release of caspase activators, such as cytochrome *c*, into the cytoplasm. Treating HL-60 cells with **11** at 10 µM for 6 h had no significant effect on cytosolic cytochrome *c* levels (Figure 7). Taken together, these results suggested that **11** exerts apoptotic effects in HL-60 cells through a mitochondria-independent pathway.

## 3. Material and Methods

### 3.1. General Experimental Procedures 

Optical rotations were measured using a JASCO P-1030 (Tokyo, Japan) automatic digital polarimeter. Ultraviolet and IR spectra were recorded on a JASCO V-630 UV-Vis spectrophotometer and on a JASCO FT-IR 410 spectrophotometer, respectively. NMR spectroscopic data were obtained on a DRX-500 (500 MHz for ^1^H-NMR) or a DPX-600 (600 MHz for ^1^H-NMR) spectrometer using standard Bruker pulse programs at 300 K (Bruker, Karlsruhe, Germany). Chemical shifts were given as δ with reference to tetramethylsilane as an internal standard. Please see Supplementary Materials for the NMR spectra. HRESIMS data were obtained on a Waters-Micromass LCT mass spectrometer (Manchester, UK). CC was performed using porous-polymer polystyrene resin Diaion HP-20 (50 mesh, Mitsubishi-Chemical, Tokyo, Japan), silica gel Chromatorex BW-300 (300 mesh, Fuji-Silysia Chemical, Aichi, Japan), and ODS silica gel COSMOSIL 75C_18_-OPN (75 μM particle size, Nacalai Tesque, Kyoto, Japan). Thin-layer chromatography (TLC) was conducted on precoated silica gel 60 F_254_ or RP_18_ F_254_S plates (0.25-mm thick, Merck, Darmstadt, Germany), and the spots were detected by spraying the plates with 10% H_2_SO_4_ aqueous solution, followed by heating. HPLC was performed with a system consisting of a CCPM pump (Shimadzu, Kyoto, Japan), an RI-8021 (Tosoh, Tokyo, Japan) or a Shodex OR-2 (Showa-Denko, Tokyo, Japan) detector, and a Rheodyne injection port (Rohnert Park, CA, USA). A TSK gel ODS-100Z column (10 mm i.d. × 250 mm, 5 µm, Tosoh, Tokyo, Japan) was used for preparative HPLC. The following materials and biochemical-grade reagents were used for the cell culture assays: a Spectra Classic microplate reader (Tecan, Salzburg, Austria); a 96-well flat-bottom plate (Iwaki Glass, Chiba, Japan); HL-60 cells (JCRB 0085) and A549 cells (JCRB 0076) (Human Science Research Resources Bank, Osaka, Japan); fetal bovine serum (FBS) (Bio-Whittaker, Walkersville, MD, USA); 0.25% trypsin-ethylenediaminetetraacetic acid (EDTA) solution, RPMI-1640 medium, minimum essential medium (MEM), Triton X, cisplatin, etoposide and MTT (Sigma, St. Louis, MO, USA); penicillin G sodium salt and streptomycin sulfate (Gibco, Gland Island, NY, USA); ribonuclease A (RNase), paraformaldehyde and phosphate-buffered saline (PBS) (Wako Pure Chemical Industries, Osaka, Japan); and propidium iodide (PI) (Molecular Probes, Eugene, OK, USA).

### 3.2. Plant Material

The fresh bulbs of *O. saundersiae* were obtained from Sakata Seed Corporation (Kanagawa, Japan) in 2013. A voucher specimen has been deposited at the herbarium of this university (KS-2013-006).

### 3.3. Extraction and Isolation

The fresh bulbs of *O. saundersiae* (9.7 kg) were extracted with MeOH (3 × 18 L). After evaporating the solvent in vacuo, the MeOH extract (510 g) was applied to a Diaion HP-20 column (80 mm i.d. × 470 mm) and successively separated by MeOH/H_2_O (1:4), EtOH, and EtOAc (each 3 L). The EtOH-eluate (75.0 g) was subjected to silica gel (100 mm i.d. × 490 mm) and eluted with a stepwise gradient mixture of CHCl_3_/MeOH (9:1; 4:1; 2:1), and finally with MeOH alone, to yield 10 subfractions (A–J). Fraction C (11.7 g) was separated by an ODS silica gel column (75 mm i.d. × 350 mm) and eluted sequentially with MeCN/H_2_O (2:3) into 10 subfractions (C.1–C.10). Fraction C.5 was passed through an ODS silica gel column (50 mm i.d. × 300 mm) using MeCN/H_2_O (2:3) to afford fractions C.5.1–C.5.5. Fraction C.5.3 (485 mg) was further divided by silica gel CC (35 mm i.d. × 390 mm) and eluted with CHCl_3_/MeOH (9:1) into fractions C.5.3.1–C.5.3.15. Fraction C.5.3.12 (142 mg) was purified by preparative HPLC using MeOH/H_2_O (3:2) to yield **14** (20.8 mg). Fraction C.5.5 (1.03 g) was separated by a silica gel column (50 mm i.d. × 300 mm) and eluted with CHCl_3_/MeOH (9:1) and MeOH into fractions C.5.5.1–C.5.5.9. Fraction C.5.5.2 (276 mg) was subjected to ODS silica gel CC (43 mm i.d. × 320 mm) and eluted with MeOH/H_2_O (7:3) to yield **19** (127 mg). Fraction C.5.5.3 (567 mg) was further separated by ODS silica gel CC (60 mm i.d. × 250 mm) and eluted with MeOH/H_2_O (7:3) and MeOH into fractions C.5.5.3.1–C.5.5.3.18. Fraction C.5.5.3.8 (133 mg) was purified by ODS silica gel CC (43 mm i.d. × 275 mm) using MeCN/H_2_O (9:11) to yield **15** (64.2 mg). Fraction C.5.5.3.13 (19.1 mg) was subjected to silica gel CC (28 mm i.d. × 225 mm) and eluted with CHCl_3_/MeOH/H_2_O (19:1:0.1) to yield **11** (12.7 mg). Fraction C.5.5.4 (84.2 mg) was chromatographed on ODS silica gel (60 mm i.d. × 265 mm) and eluted with MeOH/H_2_O (7:3) to yield **12** (8.6 mg). Fraction C.5.5.5 (145 mg) was subjected to CC (43 mm i.d. × 335 mm) on ODS silica gel, eluted with MeCN/H_2_O (2:3) and MeOH/H_2_O (7:3), to afford **4** (11.5 mg). Fraction C.8 (4.17 g) was subjected to CC (65 mm i.d. × 490 mm) on silica gel, eluted with CHCl_3_/MeOH (6:1), followed by preparative HPLC using MeOH/H_2_O (7:3) to afford **16** (15.3 mg) and **17** (2.6 mg). Fraction D (6.61 g) was separated by ODS silica gel CC (90 mm i.d. × 100 mm) using MeCN/H_2_O (1:2) to nine subfractions (D.1–D.9). Fraction D.2 (584 mg) was subjected to an ODS silica gel column (50 mm i.d. × 300 mm), using MeCN/H_2_O (1:3) as the eluent, and preparative HPLC using MeOH/H_2_O (3:2) to afford **18** (23.9 mg). Fraction D.3 (670 mg) was subjected to an ODS silica gel column, using MeCN/H_2_O (2:5; 7:13) and MeOH/H_2_O (3:2) as the eluents, and a silica gel column with CHCl_3_–MeOH (7:1) to afford **13** (55.3 mg). Fraction D.5 (242 mg) was separated using ODS silica gel CC and eluted with MeOH/H_2_O (3:2) into fractions D.5.1–D.5.8. Fraction D.5.3 (100 mg) was subjected to a silica gel column and eluted with CHCl_3_/MeOH/H_2_O (5:1:0:1; 19:1:0.1) to yield **5** (38.4 mg). Fraction D.6 (317 mg) was separated using ODS silica gel CC and eluted with MeOH/H_2_O (3:2) into fractions D.6.1–D.6.7. Fraction D.6.2 (68.6 mg) was added to an ODS silica gel column (40 mm i.d. × 320 mm), with MeCN/H_2_O (3:7) as the eluent, and a silica gel column with CHCl_3_/MeOH/H_2_O (8:1:0.1) to yield **6** (23.7 mg). Fraction D.9 (4.15 g) was separated using ODS silica gel CC (40 mm i.d. × 300 mm) and eluted with MeOH/H_2_O (7:3) into fractions D.9.1–D.9.19. Fraction D.9.4 (14.3 mg) and D.9.7 (108 mg) were subjected to preparative HPLC using MeCN/H_2_O (2:3) to yield **2** (4.1 mg) and 8 (15.6 mg), respectively. Fraction D.9.8 (161 mg) was subjected to ODS silica gel CC (34 mm i.d. × 410 mm) and eluted with MeCN/H_2_O (11:9) to yield fractions D.9.8.1–D.9.8.10. Fraction D.9.8.2 (77.4 mg) was further divided by CC (38 mm i.d. × 360 mm) on ODS silica gel and eluted with MeOH/H_2_O (7:3) into fractions D.9.8.2.1–D.9.8.2.8. Purification of fraction D.9.8.2.3 (20.1 mg) by preparative HPLC using MeCN/H_2_O (2:3) led to the isolation of **7** (2.52 mg). Compound **3** (15.2 mg) was obtained from fraction D.9.8.2.5 (18.3 mg) by subjecting it to ODS silica gel CC (35 mm i.d. × 340 mm) using MeCN/H_2_O (2:3). Fraction D.9.12 (150 mg) was subjected to an ODS silica gel column (38 mm i.d. × 340 mm) with MeCN/H_2_O (2:3) and MeOH/H_2_O (7:3) as the eluents, and a silica gel column with CHCl_3_/MeOH (6:1) to afford **10** (6.5 mg). Fraction F (7.9 g) was subjected to ODS silica gel CC (70 mm i.d. × 320 mm) and eluted with MeOH/H_2_O (3:2) to yield 10 subfractions (F.1–F.10). Fraction F.4 (375 mg) was subjected to preparative HPLC using MeCN/H_2_O (1:2) to yield 9 (4.8 mg), and **1** with minor impurities, which was purified by ODS silica gel CC (20 mm i.d. × 235 mm) using MeOH/H_2_O (1:1) to furnish pure **1** (10.9 mg).

### 3.4. Structural Characterization

Compound **1**: Amorphous solid; ${\left[\alpha \right]}_{D}^{25}$ −7.06 (*c* = 0.05, MeOH); IR (film) ν_max_: 3393 (OH), 2927 (CH), 1729 (C=O) cm^−1^; HRESIMS *m*/*z*: 807.4518 [M + Na]^+^ (calcd. for C_41_H_68_O_14_Na: 807.4507). For ^1^H-NMR spectral data of the sugar and acyl moieties, see Table 1. For ^13^C-NMR spectral data, see Table 3. ^1^H-NMR spectral data of the aglycone moiety (C_5_D_5_N) δ_H_: 5.44 (1H, br d, *J* = 4.9 Hz, H-6), 4.41 (1H, m, H-16), 4.26 (1H, m, H-11), 4.06 (1H, m, H-22), 4.04 (1H, m, H-3), 1.31 (3H, s, Me-19), 1.23 (3H, d, *J* = 6.8 Hz, Me-21), 1.05 (3H, d, *J* = 6.3 Hz, Me-26), 1.03 (3H, d, *J* = 6.3 Hz, Me-27), 0.99 (3H, s, Me-18).

Compound **2**: Amorphous solid; ${\left[\alpha \right]}_{D}^{25}$ −34.8 (*c* = 0.05, MeOH); IR (film) ν_max_: 3362 (OH), 2931 (CH), 1746 (C=O) cm^−1^; HRESIMS *m*/*z*: 849.4618 [M + Na]^+^ (calcd. for C_43_H_70_O_15_Na: 849.4612). For ^1^H-NMR spectral data of the sugar and acyl moieties, see Table 1. For ^13^C-NMR spectral data, see Table 3. ^1^H-NMR spectral data of the aglycone moiety (C_5_D_5_N) δ_H_: 5.40 (1H, overlapping with water signal, H-6), 4.40 (1H, m, H-16), 4.21 (1H, m, H-11), 4.00 (1H, m, H-3), 3.98 (1H, m, H-22), 1.24 (3H, s, Me-19), 1.18 (3H, d, *J* = 6.9 Hz, Me-21), 1.04 (3H, d, *J* = 6.6 Hz, Me-27), 0.98 (3H, d, *J* = 6.6 Hz, Me-26), 0.94 (3H, s, Me-18).

Compound **3**: Amorphous solid; ${\left[\alpha \right]}_{D}^{25}$ −21.5 (*c* = 0.10, MeOH); UV λ_max_ (MeOH) nm (log ε): 257 (4.12), 206 (4.14); IR (film) ν_max_: 3389 (OH), 2932 (CH), 1748 (C=O), 1606, 1512, 1463 (aromatic ring) cm^−1^; HRESIMS *m*/*z*: 941.4871 [M + Na]^+^ (calcd. for C_49_H_74_O_16_Na: 941.4875). For ^1^H-NMR spectral data of the sugar and acyl moieties, see Table 1. For ^13^C-NMR spectral data, see Table 3. ^1^H-NMR spectral data of the aglycone moiety (C_5_D_5_N) δ_H_: 5.42 (1H, br d, *J* = 4.0 Hz, H-6), 4.46 (1H, m, H-16), 4.25 (1H, m, H-11), 4.05 (1H, m, H-22), 4.02 (1H, m, H-3), 1.28 (3H, s, Me-19), 1.23 (3H, d, *J* = 5.3 Hz, Me-21), 0.99 (3H, d, *J* = 6.4 Hz, Me-27), 0.99 (3H, s, Me-18), 0.95 (3H, d, *J* = 6.4 Hz, Me-26).

Compound **4**: Amorphous solid; ${\left[\alpha \right]}_{D}^{25}$ −19.7 (*c* = 0.10, MeOH); UV λ_max_ (MeOH) nm (log ε): 262 (3.91), 257 (3.91), 215 (4.25); IR (film) ν_max_: 3432 (OH), 2926 (CH), 1732 and 1716 (C=O), 1616, 1508, 1460 (aromatic ring) cm^−1^; HRESIMS *m*/*z*: 1001.5087 [M + Na]^+^ (calcd. for C_51_H_78_O_18_Na: 1001.5086). For ^1^H-NMR spectral data of the sugar and acyl moieties, see Table 1. For ^13^C-NMR spectral data, see Table 3. ^1^H-NMR spectral data of the aglycone moiety (C_5_D_5_N) δ_H_: 5.44 (1H, br d, *J* = 5.4 Hz, H-6), 4.48 (1H, m, H-16), 4.26 (1H, m, H-11), 4.07 (1H, m, H-22), 4.00 (1H, m, H-3), 1.29 (3H, s, Me-19), 1.25 (3H, d, *J* = 7.0 Hz, Me-21), 1.01 (3H, d, *J* = 6.5 Hz, Me-27), 1.00 (3H, s, Me-18), 0.96 (3H, d, *J* = 6.5 Hz, Me-26).

Compound **5**: Amorphous solid; ${\left[\alpha \right]}_{D}^{25}$ 70.6 (*c* = 0.10, MeOH); IR (film) ν_max_: 3361 (OH), 2925 (CH), 1719 (C=O) cm^−1^; HRESIMS *m*/*z*: 643.3820 [M + Na]^+^ (calcd. for C_35_H_56_O_9_Na: 643.3822). For ^1^H-NMR spectral data of the sugar and acyl moieties, see Table 1. For ^13^C-NMR spectral data, see Table 3. ^1^H-NMR spectral data of the aglycone moiety (C_5_D_5_N) δ_H_: 5.82 (1H, br t, *J* = 7.2 Hz, H-24), 5.65 (1H, br d, *J* = 4.7 Hz, H-6), 4.42 (1H, m, H-16), 4.28 (1H, m, H-11), 4.22 (1H, m, H-22), 3.91 (1H, m, H-3), 1.78 (3H, s, Me-27), 1.73 (3H, s, Me-26), 1.41 (3H, s, Me-19), 1.26 (3H, d, *J* = 6.8 Hz, Me-21), 1.00 (3H, s, Me-18).

Compound **6**: Amorphous solid; ${\left[\alpha \right]}_{D}^{25}$ −27.2 (*c* = 0.098, MeOH); IR (film) ν_max_: 3389 (OH), 1747 (C=O), 1449 (C=C) cm^−1^; HRESIMS *m*/*z*: 847.4457 [M + Na]^+^ (calcd. for C_43_H_68_O_15_Na: 847.4456). For ^1^H-NMR spectral data of the sugar and acyl moieties, see Table 1. For ^13^C-NMR spectral data, see Table 3. ^1^H-NMR spectral data of the aglycone moiety (C_5_D_5_N) δ_H_: 5.77 (1H, dd, *J* = 7.2, 3.2 Hz, H-24), 5.41 (1H, br d, *J* = 4.4 Hz, H-6), 4.41 (1H, m, H-16), 4.23 (1H, m, H-11), 4.12 (1H, m, H-22), 4.01 (1H, m, H-3), 1.77 (3H, s, Me-26), 1.73 (3H, s, Me-27), 1.26 (3H, s, Me-19), 1.22 (3H, d, *J* = 7.2 Hz, Me-21), 0.94 (3H, s, Me-18).

Compound **7**: Amorphous solid; ${\left[\alpha \right]}_{D}^{25}$ −11.5 (*c* = 0.042, MeOH); UV λ_max_ (MeOH) nm (log ε): 256 (4.17), 205 (4.56); IR (film) ν_max_: 3389 (OH), 2922 (CH), 1746 and 1724 (C=O), 1606, 1512, 1462 (aromatic ring) cm^−1^; HRESIMS *m*/*z*: 939.4721 [M + Na]^+^ (calcd. for C_49_H_72_O_16_Na: 939.4718). For ^1^H-NMR spectral data of the sugar and acyl moieties, see Table 2. For ^13^C-NMR spectral data, see Table 3. ^1^H-NMR spectral data of the aglycone moiety (C_5_D_5_N) δ_H_: 5.78 (1H, m, H-24), 5.43 (1H, br d, *J* = 3.9 Hz, H-6), 4.46 (1H, m, H-16), 4.24 (1H, ddd, *J* = 10.0, 10.0, 4.8 Hz, H-11), 4.18 (1H, m, H-22), 4.02 (1H, m, H-3), 1.71 (3H, s, Me-26), 1.70 (3H, s, Me-27), 1.27 (3H, s, Me-19), 1.26 (3H, d, *J* = 6.9 Hz, Me-21), 0.98 (3H, s, Me-18).

Compound **8**: Amorphous solid; ${\left[\alpha \right]}_{D}^{25}$ −21.7 (*c* = 0.10, MeOH); UV λ_max_ (MeOH) nm (log ε): 267 (3.92), 214 (4.38); IR (film) ν_max_: 3389 (OH), 2936 (CH), 1724 (C=O), 1590, 1504, 1455 (aromatic ring) cm^−1^; HRESIMS *m*/*z*: 999.4930 [M + Na]^+^ (calcd. for C_51_H_76_O_18_Na: 999.4929). For ^1^H-NMR spectral data of the sugar and acyl moieties, see Table 2. For ^13^C-NMR spectral data, see Table 3. ^1^H-NMR spectral data of the aglycone moiety (C_5_D_5_N) δ_H_: 5.79 (1H, dd, *J* = 6.5, 6.5 Hz, H-24), 5.43 (1H, br d, *J* = 4.6 Hz, H-6), 4.48 (1H, m, H-16), 4.24 (1H, ddd, *J* = 10.3, 10.3, 4.3 Hz, H-11), 4.18 (1H, m, H-22), 4.03 (1H, m, H-3), 1.72 (3H, s, Me-26), 1.70 (3H, s, Me-27), 1.28 (3H, s, Me-19), 1.27 (3H, d, *J* = 7.0 Hz, Me-21), 0.98 (3H, s, Me-18).

Compound **9**: Amorphous solid; ${\left[\alpha \right]}_{D}^{25}$ −33.9 (*c* = 0.05, MeOH); IR (film) ν_max_: 3412 (OH), 2925 (CH), 1730 (C=O) cm^−1^; HRESIMS *m*/*z*: 993.5050 [M + Na]^+^ (calcd. for C_49_H_78_O_19_Na: 993.5035). For ^1^H-NMR spectral data of the sugar and acyl moieties, see Table 2. For ^13^C-NMR spectral data, see Table 3. ^1^H-NMR spectral data of the aglycone moiety (C_5_D_5_N) δ_H_: 5.79 (1H, m, H-24), 5.41 (1H, br d, *J* = 5.2 Hz, H-6), 4.44 (1H, m, H-16), 4.25 (1H, m, H-11), 4.14 (1H, m, H-22), 3.90 (1H, m, H-3), 1.79 (3H, s, Me-26), 1.75 (3H, s, Me-27), 1.45 (3H, s, Me-19), 1.24 (3H, d, *J* = 6.8 Hz, Me-21), 0.96 (3H, s, Me-18). 

Compound **10**: Amorphous solid; ${\left[\alpha \right]}_{D}^{25}$ −42.6 (*c* = 0.08, MeOH); IR (film) ν_max_: 3391 (OH), 2929 (CH), 1748 (C=O) cm^−1^; HRESIMS *m*/*z*: 833.4661 [M + Na]^+^ (calcd. for C_43_H_70_O_14_Na: 833.4663). For ^1^H-NMR spectral data of the sugar and acyl moieties, see Table 2. For ^13^C-NMR spectral data, see Table 3. ^1^H-NMR spectral data of the aglycone moiety (C_5_D_5_N) δ_H_: 5.33 (1H, d, *J* = 4.9 Hz, H-6), 4.40 (1H, m, H-16), 4.02 (1H, m, H-22), 3.95 (1H, m, H-3), 1.23 (3H, d, *J* = 6.9 Hz, Me-21), 1.09 (3H, d, *J* = 6.6 Hz, Me-27), 1.03 (3H, d, *J* = 6.6 Hz, Me-26), 0.94 (3H, s, Me-19), 0.90 (3H, s, Me-18).

Compound **11**: Amorphous solid; ${\left[\alpha \right]}_{D}^{25}$ −27.7 (*c* = 0.10, MeOH); IR (film) ν_max_: 3376 (OH), 2932 (CH), 1448 (C=C) cm^−1^; HRESIMS *m*/*z*: 585.3765 [M + Na]^+^ (calcd. for C_33_H_54_O_8_Na: 585.3767). For ^1^H-NMR spectral data of the sugar and acyl moieties, see Table 2. For ^13^C-NMR spectral data, see Table 3. ^1^H-NMR spectral data of the aglycone moiety (C_5_D_5_N) δ_H_: 5.56 (1H, m, H-24), 5.40 (1H, br d, *J* = 4.1 Hz, H-6), 4.41 (1H, ddd, *J* = 8.0, 8.0, 4.0 Hz, H-16), 4.13 (1H, m, H-22), 3.85 (1H, m, H-3), 1.70 (3H, s, Me-27), 1.67 (3H, s, Me-26), 1.27 (3H, d, *J* = 6.9 Hz, Me-21), 1.08 (3H, s, Me-19), 0.96 (3H, s, Me-18).

Compound **12**: Amorphous solid; ${\left[\alpha \right]}_{D}^{25}$ −18.6 (*c* = 0.10, MeOH); IR (film) ν_max_: 3359 (OH), 2930 (CH), 1747 (C=O) cm^−1^; HRESIMS *m*/*z*: 831.4511 [M + Na]^+^ (calcd. for C_43_H_68_O_14_Na: 831.4507). For ^1^H-NMR spectral data of the sugar and acyl moieties, see Table 2. For ^13^C-NMR spectral data, see Table 3. ^1^H-NMR spectral data of the aglycone moiety (C_5_D_5_N) δ_H_: 5.79 (1H, m, H-24), 5.62 (1H, overlapping with water signal, H-6), 4.40 (1H, m, H-16), 4.15 (1H, m, H-22), 3.94 (1H, m, H-3), 1.79 (3H, s, Me-26), 1.75 (3H, s, Me-27), 1.24 (3H, d, *J* = 6.9 Hz, Me-21), 0.91 (3H, s, Me-19), 0.87 (3H, s, Me-18).

Catalyti hydrogenation of **12**: Compound **12** (2.0 mg) was dissolved in MeOH (2 mL) and reduced with 10% Pd–C (15 mg) under a H_2_ atmosphere for 4 h at room temperature. The reaction mixture, after removal of the catalyst by filtration, was subsequently passed through a Sep-Pak C18 cartridge (Waters, Milford, MA) and then chromatographed on ODS silica gel, using MeOH/H_2_O (7:3) as an eluent, to yield **10** (1.33 mg).

Alkaline methanolysis of **1**–**8** and **10**: Compounds **1**–**2**, **4**–**5** and **10** (each 1.0 mg), **3** (5.0 mg), **6** (10.0 mg), **7** (2.52 mg), and **8** (6.32 mg), were separately treated with 3% NaOMe in MeOH (2.0 mL) at room temperature for 1 h. The reaction mixture was neutralized by passing it through an Amberlite IR120 (Organo, Tokyo, Japan) column and then chromatographed on silica gel, using CHCl_3_/MeOH/H_2_O (9:1:0; 5:1:0.1; 4:1:0; 3:1:0.1) as an eluent, to yield **18** (0.54 mg) from **5**, **1a** (0.8 mg) from **1**, **1a** (0.72 mg) from **2**, **1a** (2.24 mg) and methyl *p*-methoxybenzoate (1.17 mg) from **3**, **1a** (0.65 mg) and methyl 3,4,5-trimethoxybenzoate (0.15 mg) from **4**, **6a** (6.15 mg) from **6**, **6a** (1.73 mg) and methyl *p*-methoxybenzoate (0.28 mg) from **7**, **6a** (3.88 mg) and methyl 3,4,5-trimethoxybenzoate (0.90 mg) from **8**, and **10a** (0.71 mg) from **10**. 

Compound **1a**: Amorphous solid; ${\left[\alpha \right]}_{D}^{25}$ −33.9 (*c* = 0.05, MeOH); IR (film) ν_max_: 3390 (OH), 2921 (CH) cm^−1^; HRESIMS *m*/*z*: 765.4369 [M + Na]^+^ (calcd. for C_39_H_66_O_13_Na: 765.4401). For ^1^H-NMR spectral data of the sugar and acyl moieties, see Table 1. For ^13^C-NMR spectral data, see Table 3. ^1^H-NMR spectral data of the aglycone moiety (C_5_D_5_N)δ_H_: 5.43 (1H, br d, *J* = 5.0 Hz, H-6), 4.43 (1H, m, H-16), 4.29 (1H, m, H-11), 4.40 (1H, m, H-22), 3.99 (1H, m, H-3), 1.30 (3H, s, Me-19), 1.21 (3H, d, *J* = 6.8 Hz, Me-21), 1.01 (3H, s, Me-18), 0.83 (3H, d, *J* = 6.6 Hz, Me-26), 0.82 (3H, d, *J* = 6.6 Hz, Me-27). 

Acid hydrolysis of **1a**: Compound **1a** (3.2 mg) was dissolved in 1 M HCl (dioxane/H_2_O, 1:1, 2.0 mL) and heated at 95 °C for 1 h under an Ar atmosphere. After cooling, the reaction mixture was neutralized via passage through an Amberlite IRA-96 column (Organo) and then chromatographed on silica gel, using CHCl_3_/MeOH (19:1) as an eluent, to yield (22*S*)-cholest-5-ene-3β,11α,16β,22-tetrol (0.5 mg) [7] and a sugar fraction (1.3 mg). The sugar fraction was analyzed using HPLC under the following conditions: column, Capcell Pak NH_2_ UG80 (4.6 mm i.d. × 250 mm, 5 μm, Shiseido, Tokyo, Japan); solvent, MeCN/H_2_O (17:3); detection, refractive index (RI) and optical rotation (OR); flow rate, 1.0 mL/min. HPLC analysis of the sugar fraction showed the presence of l-rhamnose and d-glucose; *t*_R_ (min): 7.38 (l-rhamnose, negative optical rotation), 14.0 (d-glucose, positive optical rotation).

Compound **6a**: Amorphous solid; ${\left[\alpha \right]}_{D}^{25}$ −33.9 (*c* = 0.05, MeOH); IR (film) ν_max_: 3390 (OH), 2921 (CH) cm^−1^; HRESIMS *m*/*z*: 765.4369 [M + Na]^+^ (calcd. for C_39_H_66_O_13_Na: 765.4401). For ^1^H-NMR spectral data of the sugar and acyl moieties, see Table 2. For ^13^C-NMR spectral data, see Table 3. ^1^H-NMR spectral data of the aglycone moiety (C_5_D_5_N) δ_H_: 5.53 (1H, br t, *J* = 7.3 Hz, H-24), 5.44 (1H, br d, *J* = 5.3 Hz, H-6), 4.42 (1H, m, H-16), 4.26 (1H, m, H-11), 4.12 (1H, m, H-22), 4.04 (1H, m, H-3), 1.69 (3H, s, Me-27), 1.67 (3H, s, Me-26), 1.31 (3H, s, Me-19), 1.25 (3H, d, *J* = 6.9 Hz, Me-21), 1.01 (3H, s, Me-18).

Enzymatic hydrolysis of **6a**: Compound **6a** (3.0 mg) was treated with β-d-glucosidase (10 mg) in AcOH/AcONa buffer (pH 5.0, 3.0 mL) at room temperature for 47 h. The crude hydrolysate was chromatographed on silica gel and eluted with CHCl_3_/MeOH/H_2_O (9:1:0.1), followed by MeOH, to yield **18** (1.7 mg) and a sugar fraction (0.6 mg). The glucose present in the sugar fraction was identified under the same conditions as in the case of **2a**. *t*_R_ (min): 14.0 (d-glucose, positive optical rotation).

Acid hydrolysis of **9**: A solution of **9** (1.0 mg) in 1 M HCl (dioxane–H_2_O, 1;1, 2 mL) was heated at 95 °C for 90 min under an Ar atmosphere. After cooling, the reaction mixture was neutralized via passage through an Amberlite IRA-96 (Organo) column. The reaction mixture was chromatographed on silica gel, eluted with CHCl_3_–MeOH (4:1), to yield a sugar fraction, but genuine aglycones could not be obtained. HPLC analysis of the sugar fraction showed the presence of l-rhamnose and d-glucose; *t*_R_ (min): 8.21 (l-rhamnose, negative optical rotation), 15.1 (d-glucose, positive optical rotation).

### 3.5. Assay for Cytotoxic Activity

HL-60 cells or A549 cells were maintained in RPMI 1640 medium or MEM, respectively, containing 10% (*v*/*v*) heat-inactivated fetal bovine serum supplemented with l-glutamine, 100 unit/mL penicillin G sodium salt, and 100 μg/mL streptomycin sulfate. Cells were stored in a humidified incubator at 37 °C with 5% CO_2_. Cell growth was measured with an MTT reduction assay, as previously described [14]. The cells (HL-60 cells: 4 × 10^4^ cells/mL, A549 cells: 1 × 10^4^ cells/mL) were continuously treated with each compound for 72 h, and cell viability was measured with the MTT reduction assay. A dose–response curve was plotted for **3**, **7**–**12**, **15**–**17**, and **19**, all of which resulted in less than 50% cell growth at a concentration of 10 µM, and the exact concentration at which 50% inhibition (IC_50_) of cell growth occurred was calculated. Each assay was performed in triplicate.

### 3.6. DAPI Staining

The cells (5 × 10^5^ cells/well) were plated on coverslips in 96-well plates. After 24 h, HL-60 cells were treated with either 10 µM of **11** or 15 µM of etoposide for 24 h. The cells were fixed with 1% glutaraldehyde for 30 min at room temperature before staining with DAPI (0.5 µg/mL in PBS) at room temperature. They were then observed immediately under a CKX41 fluorescence microscope (Olympus, Tokyo, Japan).

### 3.7. Cell Cycle Distribution Analysis

The cells (5 × 10^5^ cells/well) were treated with 10 µM of **11**, or 1 µM of colchicine as a control, for 20 h. The cells were collected, centrifuged at 1000 rpm for 5 min, then washed with PBS, and centrifuged again at 3000 rpm for 5 min. The cells were fixed with 70% EtOH at −20 °C, and then treated with FxCycle^TM^PI/RNase Staining Solution (Thermo Fisher Scientific, Kanagawa, Japan). Analysis of the cell cycle distribution was performed using a FACSCanto II flow cytometer (BD Biosciences, Franklin Lakes, NJ, USA).

### 3.8. DNA Fragmentation Assay

The cells were incubated at 37 °C for 20 h with **11** (10 µM), or etoposide (15 µM), as a control. DNA was extracted with the Wizard Genomic DNA Purification Kit (Promega, Madison, WI, USA). In brief, cells were centrifuged for 5 min at 1200 rpm. The cell pellet was re-suspended in nuclei lysis solution (600 µL). Then, 4 mg/mL RNaseA solution (3 µL) was added to the cell lysate, and the solution was incubated at 37 °C for 30 min. Protein precipitation solution (200 µL) was added to the RNaseA-treated cell lysate, after which the mixture was incubated for 5 min on ice, and then centrifuged at 15,000 rpm for 5 min. The supernatant was transferred to a clean 1.5 mL microcentrifuge tube containing isopropyl alcohol (600 µL), and was mixed by inversion. After centrifugation at 15,000 rpm for 5 min, DNA was visible as a small white pellet, which was washed with ethanol/water (7:3, *v*/*v*). Finally, the pellet was re-suspended in DNA rehydration solution (25 µL) and incubated at 65 °C for 1 h. The sample (8 µL) was subjected to 2% agarose gel electrophoresis in 40 mM Tris/acetate buffer (pH 7.4) at 50 V for 1 h. A DNA molecular weight marker (pHY marker, Takara, Shiga, Japan) and DNA from HL-60 cells that underwent etoposide (15 µM)-induced apoptosis, were used for calibration. The DNA fragmentation pattern was examined in photographs taken under ultraviolet illumination.

### 3.9. Caspase-3 Activation Assay

The activity of caspase-3 was measured using the Appocyto Caspase-3 Colorimetric Assay Kit (MBl, Aichi, Japan). HL-60 cells were treated with either 10 µM of **11** or 15 µM of the control reagent etoposide for 24 h, and the cells were then centrifuged at 400× *g* for 5 min and collected. Cell pellets were suspended in ice-cold cell lysis buffer (160 µL), and incubated on ice for 10 min. This cell pellet suspension was centrifuged at 10,000× *g* for 5 min, and the supernatant was collected. The cell lysate (50 µL, equivalent to 200 µg protein) was mixed with reaction buffer (2 × 50 µL) containing the substrate for caspase-3 (DEVD-*p*NA (*p*-nitroanilide)). After incubation for 6 h at 37 °C, the absorbance of the liberated chromophore *p*NA was measured at 405 nm in a microplate reader. The activity of caspase-3 was evaluated in triplicate.

### 3.10. Mitochondrial Membrane Potential (ΔΨm) Assay

The mitochondrial membrane potential (*ΔΨm*) was investigated using the MitoCapture™ Apoptosis Detection Kit (BioVision, Mountain View, CA, USA), according to the manufacturer’s protocol. HL-60 cells (5 × 10^5^ cells/mL) were treated with either 10 µM of **11** or 33 µM of cisplatin for 6 h, and then were centrifuged at 500 × *g* for 5 min and collected. Cell pellets were resuspended in 250 µL of MitoCapture™ solution, incubated for 15 min at 37 °C, and then centrifuged again for 5 min. Pellets were resuspended in 200 µL of incubation buffer and observed via fluorescence microscopy.

### 3.11. Release of Cytochrome C into the Cytosol

The release of cytochrome *c* into the cytosol was examined using the Cytochrome *c* Apoptosis Detection Kit (PromoKine, Heidelberg, Germany), according to the manufacturer’s protocol. HL-60 cells (2.7 × 10^7^ cells/mL) were treated with either 10 µM of **11** or 33 µM of cisplatin as a control for 6 h, and the cells were centrifuged and collected. Cells were homogenized and isolated as cytosolic and mitochondrial fractions by employing the appropriate reagents. The cytosolic and mitochondrial fractions (10 µg) were then subjected to sodium dodecyl sulfate (SDS)-polyacrylamide gel electrophoresis (PAGE). A standard western blot procedure was performed using monoclonal mouse anti-cytochrome *c* antibody, according to the manufacturer’s protocol.

### 3.12. Statistical Analysis

Data are expressed as means ± standard error of the mean (S.E.M.). Significant differences were calculated using Dunnett’s test using Excel-Toukei 2010 software (Social Survey Research Information, Osaka, Japan). *p* < 0.01 was considered statistically significant.

## 4. Conclusions

In conclusion, 19 cholestane rhamnosides were isolated from the bulbs of *O. saundersiae*, with 12 novel compounds. Compounds **10**–**12**, **16**, and **17** exhibited significant cytotoxic effects, with IC_50_ values ranging from 0.05–0.16 µM against HL-60 cells, and from 0.27–2.41 µM against A549 cells. Compound **11** showed potent cytotoxicity against HL-60 cells by inducing apoptosis. The evaluation of mitochondrial membrane potential and cytochrome *c* release into the cytoplasm revealed no differences between HL-60 cells treated with the control or compound **11**, suggesting that **11** induces apoptosis through a mitochondrion-independent apoptotic pathway.

## Figures and Tables

**Table 1:** ^1^H-NMR spectral data for the sugar and acyl Moieties of **1**–**6** and **1a** in C_5_D_5_N. ^a^

**1**					**1a**					**2**					**3**				
**Positions**		**δ_H_**		***J* (Hz)**	**Positions**		**δ_H_**		***J* (Hz)**	**Positions**		**δ_H_**		***J* (Hz)**	**Positions**		**δ_H_**		***J* (Hz)**
Glc 1		5.09	d	7.7	Glc 1		5.27	d	8.1	Glc 1		5.06	d	7.7	Glc 1		5.08	d	7.1
2		4.28	dd	9.0, 7.7	2		4.07	dd	8.1, 8.1	2		4.05	dd	8.9, 7.7	2		4.07	dd	8.8, 7.1
3		4.29	dd	9.0, 9.0	3		4.26	m		3		4.29	dd	8.9, 8.8	3		4.33	dd	8.8, 8.8
4		4.29	dd	9.0, 9.0	4		4.42	m		4		4.28	dd	8.8, 8.8	4		4.30	dd	8.8, 8.8
5		3.99	m		5		3.99	m		5		3.96	m		5		3.31	m	
6	a	4.54	dd	11.8, 2.6	6	a	4.54	dd	11.9, 2.1	6	a	4.52	dd	11.8, 2.2	6	a	4.53	dd	11.8, 2.3
	b	4.41	dd	11.8, 6.8		b	4.41	dd	11.9, 6.8		b	4.39	dd	11.8, 5.0		b	4.43	dd	11.8, 5.3
																			
Rha 1		5.05	br s		Rha 1		5.08	br s		Rha 1		5.00	br s		Rha 1		5.08	d	1.9
2		5.66	br d	2.6	2		4.48	br s		2		5.62	br d	3.3	2		5.78	dd	3.0, 1.9
3		4.54	dd	9.3, 2.6	3		4.44	dd	8.6, 3.2	3		5.76	dd	10.0, 3.3	3		6.05	dd	9.4, 3.0
4		4.19	dd	9.3, 9.3	4		4.29	m		4		4.20	dd	10.0, 10.0	4		4.38	dd	9.4, 9.4
5		4.26	m		5		4.29	m		5		4.33	dq	10.0, 6.2	5		4.42	m	
6		1.71	d	6.1	6		1.68	d	6.1	6		1.67	d	6.2	6		1.75	d	5.7
																			
Ac		2.03	s							Rha 2-*O*-Ac		2.07	s		PMBz 1		－		
										Rha 3-*O*-Ac		1.98	s		2		8.19	d	8.8
															3		6.93	d	8.8
															4		－		
															5		6.93	d	8.8
															6		8.19	d	8.8
															7		－		
															OMe		3.65	s	
																			
															Ac		2.12	s	
**4**					**5**					**6**									
**Positions**		**δ_H_**		***J* (Hz)**	**Positions**		**δ_H_**		***J* (Hz)**	**Positions**		**δ_H_**		***J* (Hz)**					
Glc 1		5.08	d	8.1	Rha 1		5.07	br s		Glc 1		5.07	d	7.7					
2		4.07	dd	8.6, 8.1	2		5.65	br d	2.2	2		4.06	dd	8.8, 7.7					
3		4.32	dd	8.9, 8.6	3		4.55	dd	9.4, 2.2	3		4.30	dd	8.8, 8.8					
4		4.29	dd	8.9, 8.9	4		4.20	dd	9.4, 9.4	4		4.29	dd	8.8, 8.8					
5		3.98	m		5		4.29	m		5		3.97	m						
6	a	4.54	dd	11.9, 2.4	6		1.71	d	6.5	6	a	4.53	dd	11.6, 2.0					
	b	4.42	dd	11.9, 6.8							b	4.41	dd	11.6, 4.9					
					Ac		2.03	s											
Rha 1		5.10	d	1.7						Rha 1		5.03	br s						
2		5.81	dd	3.3, 1.7						2		5.63	br d	3.1					
3		6.08	dd	9.6, 3.3						3		5.78	dd	9.7, 3.1					
4		4.38	dd	9.6, 9.6						4		4.21	dd	9.7, 9.7					
5		4.46	m							5		4.34	dq	9.7, 6.1					
6		1.76	d	6.0						6		1.68	d	6.1					
TMBz 1		－								Rha 2-*O*-Ac		2.09	s						
2		7.54	s							Rha 3-*O*-Ac		1.98	s						
3		－																	
4		－																	
5		－																	
6		7.54	s																
7		－																	
TMBz 3, 5-OMe		3.73	s																
TMBz 4-OMe		3.88	s																
Ac		2.17	s																

^a^ 500 MHz (**1**, **1a**, **4**, and **5**), 600 MHz (**2**, **3**, and **6**).

**Table 2:** ^1^H-NMR spectral data for the sugar and acyl Moieties of **6a** and **7**–**12** in C_5_D_5_N. ^a^

**6a**					**7**					**8**					**9**				
**Positions**		**δ_H_**		***J* (Hz)**	**Positions**		**δ_H_**		***J* (Hz)**	**Positions**		**δ_H_**		***J* (Hz)**	**Positions**		**δ_H_**		***J* (Hz)**
Glc 1		5.09	dd	7.7	Glc 1		5.08	d	7.6	Glc 1		5.09	d	7.6	Glc 1		5.07	d	7.3
2		4.07	dd	8.3, 7.7	2		4.07	dd	9.0, 7.6	2		4.07	dd	8.8, 7.6	2		4.28	dd	9.2, 7.3
3		4.31	m		3		4.33	dd	9.0, 9.0	3		4.33	dd	8.8, 8.8	3		4.31	m	
4		4.30	m		4		4.30	dd	9.0, 9.0	4		4.31	dd	8.8, 8.8	4		4.14	m	
5		3.99	m		5		3.98	m		5		3.99	m		5		4.03	m	
6	a	4.55	br d	10.8	6	a	4.54	dd	11.9, 2.2	6	a	4.55	dd	11.6, 2.7	6	a	4.49	dd	11.9, 2.3
	b	4.41	m			b	4.42	m			b	4.43	dd	11.6, 6.2		b	4.35	dd	11.9, 6.2
Rha 1		5.24	br s		Rha 1		5.10	d	1.4	Rha 1		5.12	d	1.6	Rha 1		6.39	br s	
2		4.50	br s		2		5.78	dd	2.7, 1.4	2		5.81	dd	3.1, 1.6	2		4.82	br d	3.2
3		4.43	m		3		6.08	dd	9.7, 2.7	3		6.11	dd	9.6, 3.1	3		4.67	dd	9.2, 3.2
4		4.31	m		4		4.38	dd	9.7, 9.7	4		4.39	dd	9.6, 9.6	4		4.35	m	
5		4.28	m		5		4.42	m		5		4.46	m		5		5.06	m	
6		1.68	d	5.6	6		1.75	d	5.9	6		1.76	d	6.0	6		1.75	d	6.0
					PMBz 1		－			TMBz 1		－			Rha' 1		5.07	br s	
					2		8.18	d	8.9	2		7.52	s		2		5.65	br d	3.1
					3		6.93	d	8.9	3		－			3		5.81	dd	9.9, 3.1
					4		－			4		－			4		4.23	m	
					5		6.93	d	8.9	5		－			5		4.37	m	
					6		8.18	d	8.9	6		7.52	s		6		1.75	d	6.0
					7		－			7		－							
					OMe		3.65	s		TMBz 3, 5-OMe		3.70	s		Rha' 2-*O*-Ac		2.10	s	
										TMBz 4-OMe		3.88	s		Rha' 3-*O*-Ac		1.98	s	
					Ac		2.13	s											
										Ac		2.19	s						
**10**					**11**					**12**									
**Positions**		**δ_H_**		***J* (Hz)**	**Positions**		**δ_H_**		***J* (Hz)**	**Positions**		**δ_H_**		***J* (Hz)**					
Glc 1		5.08	d	7.7	Rha 1		5.25	br s		Glc 1		5.06	d	7.7					
2		4.08	dd	8.7, 7.7	2		4.52	br d	2.8	2		4.07	dd	8.7, 7.7					
3		4.31	dd	8.7, 8.7	3		4.45	dd	8.6, 2.8	3		4.33	dd	8.7. 8.7					
4		4.31	dd	8.7, 8.7	4		4.32	dd	8.6, 8.6	4		4.29	dd	8.7, 8.7					
5		4.00	m		5		4.30	m		5		4.00	m						
6	a	4.59	dd	11.8, 2.3	6		1.67	d	5.7	6	a	4.57	dd	11.9, 2.3					
	b	4.44	dd	11.8, 5.3							b	4.42	dd	11.9, 5.3					
Rha 1		5.03	d	1.7						Rha 1		5.04	br s						
2		5.66	dd	3.3, 1.7						2		5.65	br d	3.0					
3		5.81	dd	9.7, 3.3						3		5.80	dd	9.7, 3.0					
4		4.22	dd	9.7, 9.7						4		4.22	dd	9.7, 9.7					
5		4.35	m							5		4.35	m						
6		1.70	d	6.2						6		1.69	d	6.1					
Rha 2-*O*-Ac		2.10	s							Rha 2-*O*-Ac		2.09	s						
Rha 3-*O*-Ac		2.00	s							Rha 3-*O*-Ac		1.98	s						

^a^ 500 MHz (**6a**, **9**, and **10**), 600 MHz (**7**, **8**, **11**, and **12**).

**Table 3:** ^13^C-NMR spectral data of **1**–**12**, **1a**, and **6a** in C_5_D_5_N. ^a^

Positions	1	1a	2	3	4	5	6	6a	7	8	9	10	11	12
1	39.9	39.8	39.4	39.6	39.7	40.0	39.6	39.6	39.6	39.6	39.6	37.4	37.7	37.3
2	30.5	30.4	30.2	32.1	30.5	32.2	30.4	30.5	30.4	30.4	30.4	30.3	32.6	30.2
3	78.2	78.3	78.0	78.2	78.4	71.7	78.1	78.2	78.2	78.2	78.2	78.0	71.2	78.0
4	39.7	39.6	39.6	39.8	39.9	44.1	39.8	39.9	39.8	39.8	39.7	39.3	43.4	39.2
5	141.8	141.8	141.6	141.8	142.0	142.9	141.8	141.8	141.8	141.8	141.8	141.0	142.0	140.9
6	121.5	121.5	121.2	121.4	121.4	120.8	121.4	121.5	121.4	121.4	121.5	121.7	121.1	121.7
7	32.1	32.1	31.9	30.4	32.2	32.9	32.1	32.2	32.1	32.1	32.2	32.0	32.0	31.9
8	31.7	31.6	31.4	31.5	31.7	31.8	31.5	31.7	31.6	31.6	31.7	31.8	31.9	31.8
9	57.0	56.9	56.6	56.8	57.0	57.1	56.8	57.1	56.8	56.9	56.9	50.3	50.4	50.2
10	38.8	38.8	38.6	38.7	38.9	38.8	38.7	38.8	38.7	38.8	38.9	36.9	36.9	36.8
11	68.1	68.1	67.8	68.0	68.1	68.1	68.0	68.2	68.0	68.0	68.0	21.1	21.1	21.0
12	51.7	51.7	51.4	51.6	51.8	51.8	51.6	51.7	51.7	51.7	51.7	40.0	40.0	39.9
13	42.9	42.9	42.6	42.8	42.9	43.0	42.8	42.9	42.8	42.8	42.9	42.2	42.2	42.1
14	54.4	54.4	54.1	54.3	54.4	54.4	54.2	54.4	54.2	54.3	54.3	54.9	55.0	54.7
15	35.5	35.5	35.2	35.3	35.4	35.6	35.3	35.6	35.3	35.3	35.6	35.4	35.6	35.3
16	82.7	82.3	83.0	83.2	83.3	82.9	83.2	82.2	83.2	83.2	83.4	83.0	82.0	82.9
17	57.7	57.8	57.4	57.6	57.8	57.7	57.6	57.8	57.6	57.6	57.7	57.7	57.7	57.6
18	14.3	14.3	14.0	14.2	14.3	14.4	14.2	14.4	14.2	14.2	14.2	13.1	13.2	13.0
19	19.1	19	18.8	19.0	19.1	19.3	19.0	19.1	19.0	19.0	19.1	19.4	19.6	19.3
20	35.9	35.9	35.9	36.1	36.2	35.4	35.6	35.1	35.6	35.6	38.9	36.2	35.1	35.6
21	11.9	11.8	11.8	12.0	12.0	11.9	12.0	11.8	12.0	12.0	12.0	12.1	11.8	12.1
22	72.4	72.4	72.5	72.6	72.7	71.8	72.0	71.9	72.0	72.0	72.0	72.8	72.0	72.1
23	34.6	34.3	34.3	34.5	34.6	35.4	35.4	35.3	35.4	35.5	35.5	34.6	35.3	35.4
24	36.4	36.7	36.4	36.5	36.6	123.5	123.5	123.0	123.0	123.5	123.5	36.7	123.1	123.5
25	29.0	28.6	28.8	28.9	29.0	132.3	132.2	132.4	132.2	132.3	132.2	29.0	132.3	132.2
26	23.0	22.8	22.7	22.9	22.9	26.0	25.9	25.9	25.9	25.9	26.0	22.9	25.9	26.0
27	22.9	22.7	22.6	22.8	22.9	18.3	18.1	18	18.1	18.2	18.2	23.0	18.0	18.2
	Glc	Glc	Glc	Glc	Glc	Rha	Glc	Glc	Glc	Glc	Glc	Glc	Rha	Glc
1	102.4	104.9	102.1	102.3	102.4	101.4	102.3	102.4	102.3	102.3	100.3	102.6	104.9	102.5
2	75.4	75.3	75.1	75.3	75.4	74.2	75.3	75.4	75.3	75.3	77.9	75.4	72.6	75.3
3	78.6	78.5	78.3	78.5	78.6	70.7	78.5	78.6	78.5	78.6	79.7	78.7	73.1	78.6
4	71.7	71.6	71.4	71.6	71.8	74.4	71.6	71.7	71.6	71.6	71.8	71.8	73.9	71.6
5	78.5	78.4	78.2	78.4	78.4	70.9	78.4	78.5	78.4	78.4	78.2	78.5	70.9	78.4
6	62.8	62.7	62.5	62.6	62.8	18.2	62.7	62.8	62.7	62.7	62.6	62.9	18.4	62.7
	Rha	Rha	Rha	Rha	Rha		Rha	Rha	Rha	Rha	Rha	Rha		Rha
1	101.5	102.3	101.0	101.2	101.2		101.1	104.9	101.1	101.1	101.2	101.2		101.1
2	74.2	72.6	71.1	71.4	71.5		71.4	72.6	71.6	71.6	71.5	71.4		71.4
3	70.7	73.1	72.6	73.1	73.8		72.8	73.1	73.1	73.7	72.9	72.9		72.8
4	74.1	73.9	70.6	71.1	71.3		70.9	74.0	71.2	71.3	71.0	71.0		70.9
5	71.0	70.9	70.7	71.0	71.1		70.9	71.0	71.0	71.0	71.0	71.0		70.9
6	18.3	18.4	17.9	18.2	18.2		18.1	18.4	18.2	18.2	18.2	18.2		18.1
											Rha'			
1											102.1			
2											72.6			
3											72.9			
4											74.2			
5											69.5			
6											18.7			
Ac	21.0		20.5	20.7	20.8	21.0	20.7		20.7	20.8	20.8	20.8		20.7
	170.4		170.2	169.9	170.0	170.6	170.2		170.0	170.1	170.1	170.2		170.2
Ac			20.6				20.8				20.9	20.8		20.8
			170.0				170.5				170.4	170.4		170.5
				PMBz	TMBz				PMBz	TMBz				
1				123.1	125.9				123.5	125.8				
2				132.0	107.8				132.0	107.6				
3				114.1	153.6				114.1	153.5				
4				163.8	143.3				163.8	143.0				
5				114.1	153.6				114.1	153.5				
6				132.0	107.8				132.0	107.6				
7				165.8	165.9				165.9	166.0				
OMe				55.3	56.1	(x 2)			55.4	55.9	(x 2)			
OMe					60.6					60.5				

^a^ 125 MHz (**1**, **1a**, **4**, **5**, **6a**, **9**, and **10**), 150 MHz (**2**, **3**, **6**–**8**, **11** and **12**).

**Table 4:** Cytotoxic activities of **1**–**19**, **6a**, etoposide, and cisplatin against HL-60 cells ^a^.

Compounds	IC_50_ (μM)	Compounds	IC_50_ (μM)
**1**	10<	**11**	0.16 ± 0.02
**2**	10<	**12**	0.08 ± 0.01
**3**	1.57 ± 0.10	**13**	10<
**4**	10<	**14**	10<
**5**	10<	**15**	7.12 ± 0.27
**6**	10<	**16**	0.06 ± 0.002
**6a**	10<	**17**	0.05 ± 0.01
**7**	7.72 ± 0.42	**18**	10<
**8**	5.33 ± 0.33	**19**	2.67 ± 0.31
**9**	5.94 ± 0.10	etoposide	0.23 ± 0.02
**10**	0.09 ± 0.01	cisplatin	1.52 ± 0.09

^a^ Data represent the mean value ± standard error of the mean (S.E.M.) of three experiments performed in triplicate.

**Table 5:** Cytotoxic activities of **10**–**12**, **16**, **17**, cisplatin and doxorubicin against A549 cells ^a^.

Compounds	IC_50_ (μM)	Compounds	IC_50_ (μM)
**10**	2.41 ± 0.17	**17**	0.27 ± 0.04
**11**	1.84 ± 0.31	cisplatin	2.27 ± 0.39
**12**	0.98 ± 0.09	doxorubicin	0.17 ± 0.02
**16**	0.37 ± 0.09		

^a^ Data represent the mean value ± S.E.M. of three experiments performed in triplicate.

## Supplementary Materials

The following are available online. ^1^H- and ^13^C-NMR of compounds **1**–**12**.
