# Supplementary material for: Structural Characterization of Cholestane Rhamnosides from Ornithogalum saundersiae Bulbs and Their Cytotoxic Activity against Cultured Tumor Cells
Source: Molecules. 2017 Jul 25;22(8):1243. doi: 10.3390/molecules22081243 (PMC6152286; doi:10.3390/molecules22081243)

OSN-B-FDG-h 1 1 C:\data

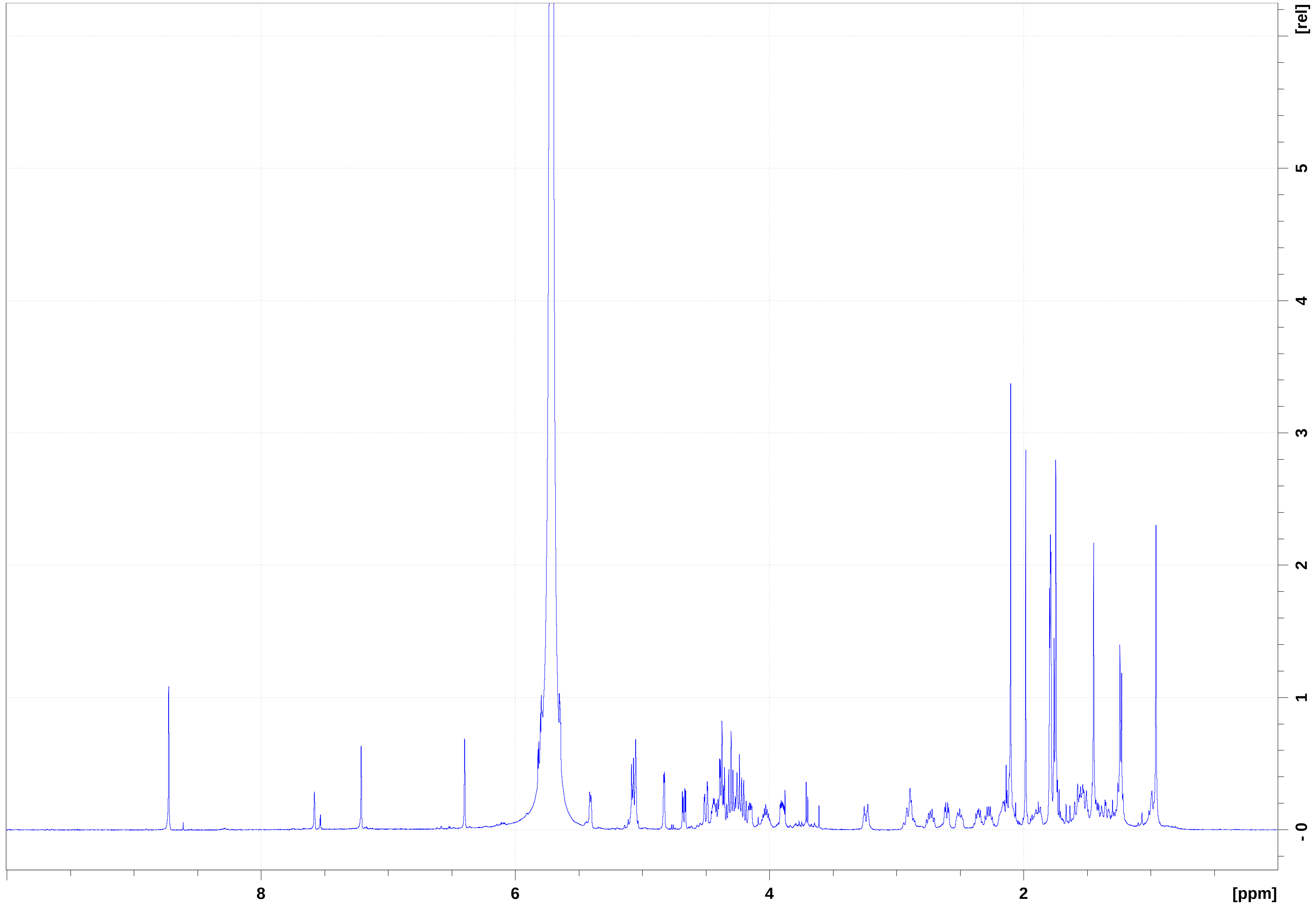

Supplement: Supplementary file 1 [file molecules-22-01243-s001.zip › Compound 9 1H-NMR.pdf]
