# Supplementary figures and images for: Structural Characterization of Cholestane Rhamnosides from Ornithogalum saundersiae Bulbs and Their Cytotoxic Activity against Cultured Tumor Cells
Source: Molecules. 2017 Jul 25;22(8):1243. doi: 10.3390/molecules22081243 (PMC6152286; doi:10.3390/molecules22081243)

OSN-B-DIHBEH 1 1 C:\data

AV-600 15/11/25

1H OSN-B-DIHBEH/C5D5N

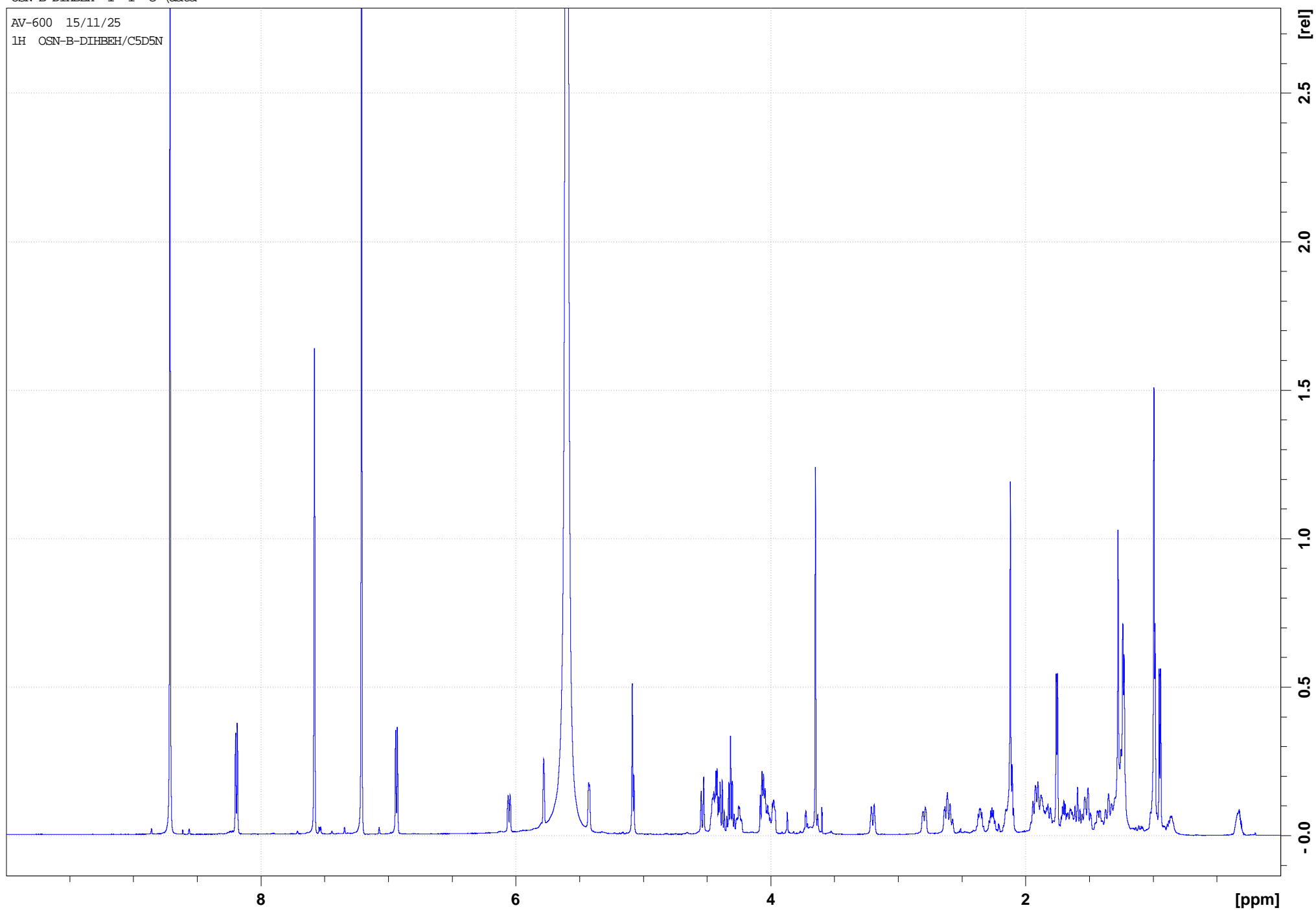

Supplement: Supplementary file 1 [file molecules-22-01243-s001.zip › Compound 3 1H-NMR.pdf]

OSN-B-DIHBEH 2 1 C:\data

AV-600 15/11/26

<sup>13</sup>C OSN-B-DIHBEH/C<sub>5</sub>D<sub>5</sub>N

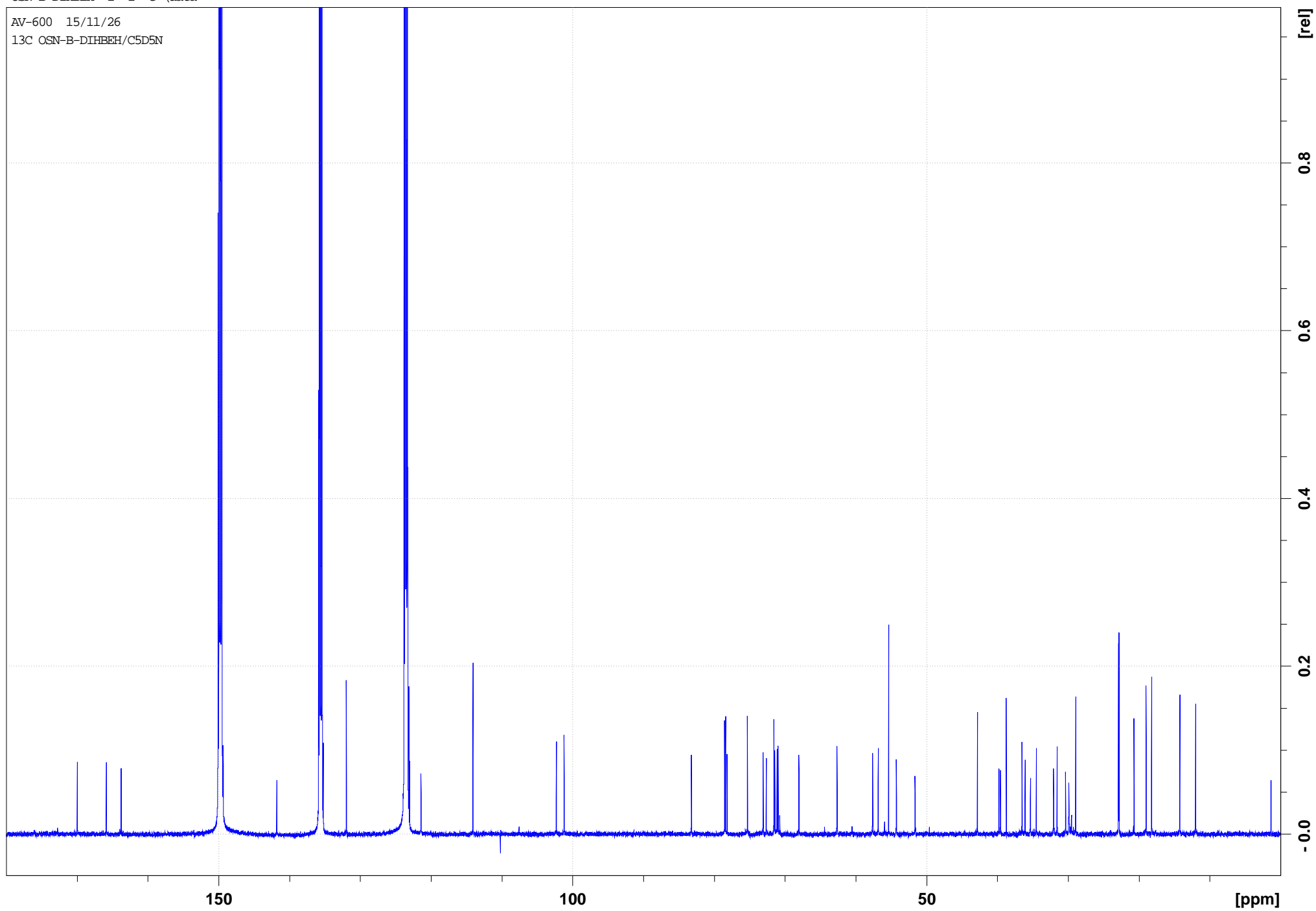

Supplement: Supplementary file 1 [file molecules-22-01243-s001.zip › Compound 3 13C-NMR.pdf]

OSN-B-DIGJGB-h 1 1 C:\data

OSN-B-DIGJGB 1H-NMR 500MHz C5D5N 2015.6.12

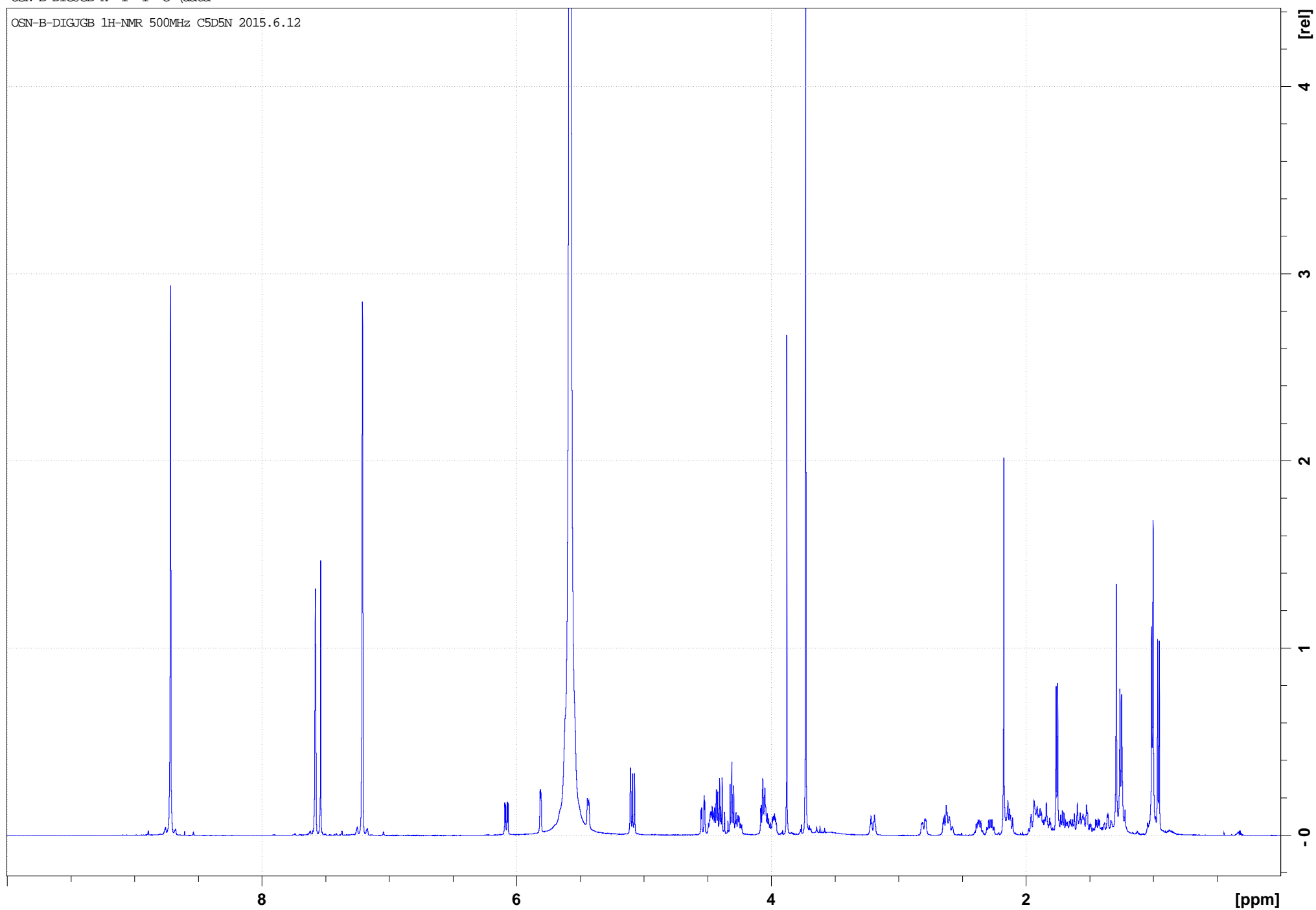

Supplement: Supplementary file 1 [file molecules-22-01243-s001.zip › Compound 4 1H-NMR.pdf]

OSN-B-DIGJGB-c 1 1 C:\data

OSN-B-DIGJGB 13C-NMR 125Mhz CSD5N 2015.6.12

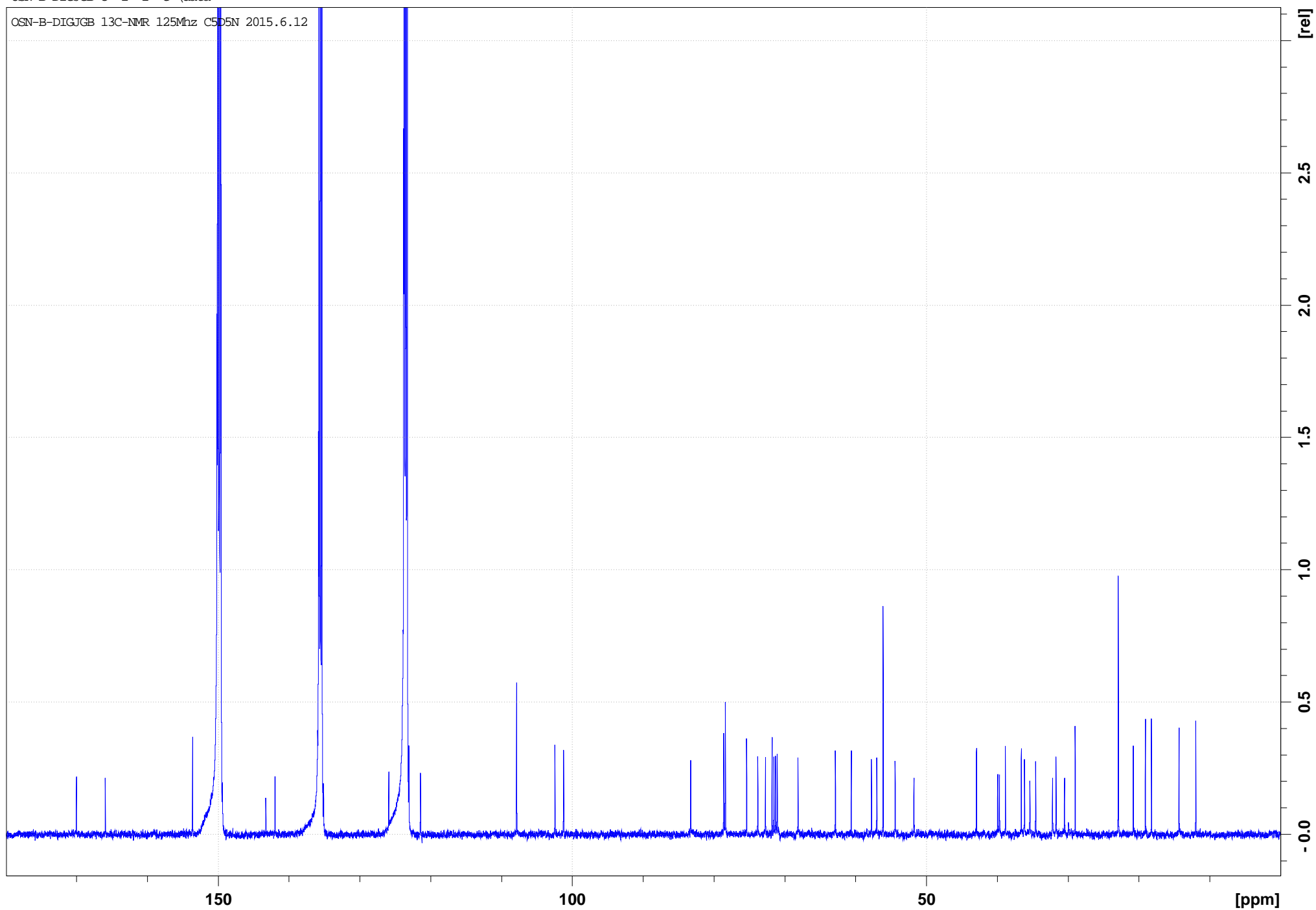

Supplement: Supplementary file 1 [file molecules-22-01243-s001.zip › Compound 4 13C-NMR.pdf]

OSN-B-CEAE-h 1 1 C:\data  
OSN-B-CEAE 1H-NMR 500MHz pyr 2015.9.21

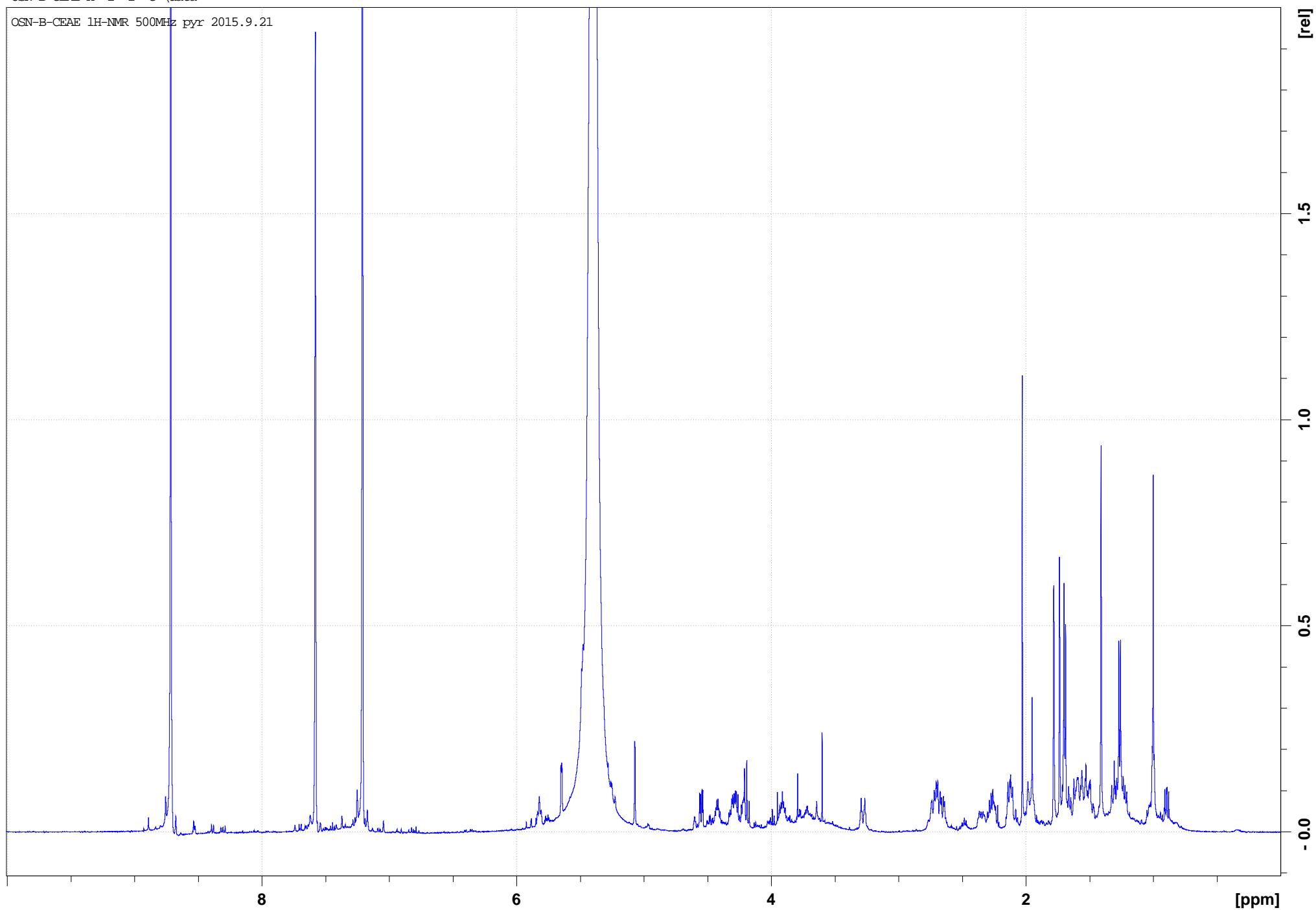

Supplement: Supplementary file 1 [file molecules-22-01243-s001.zip › Compound 5 1H-NMR.pdf]

OSN-B-CEAE-c 1 1 C:\data  
OSN-B-CEAE 13C-NMR 125MHz pyr 2015.9.22

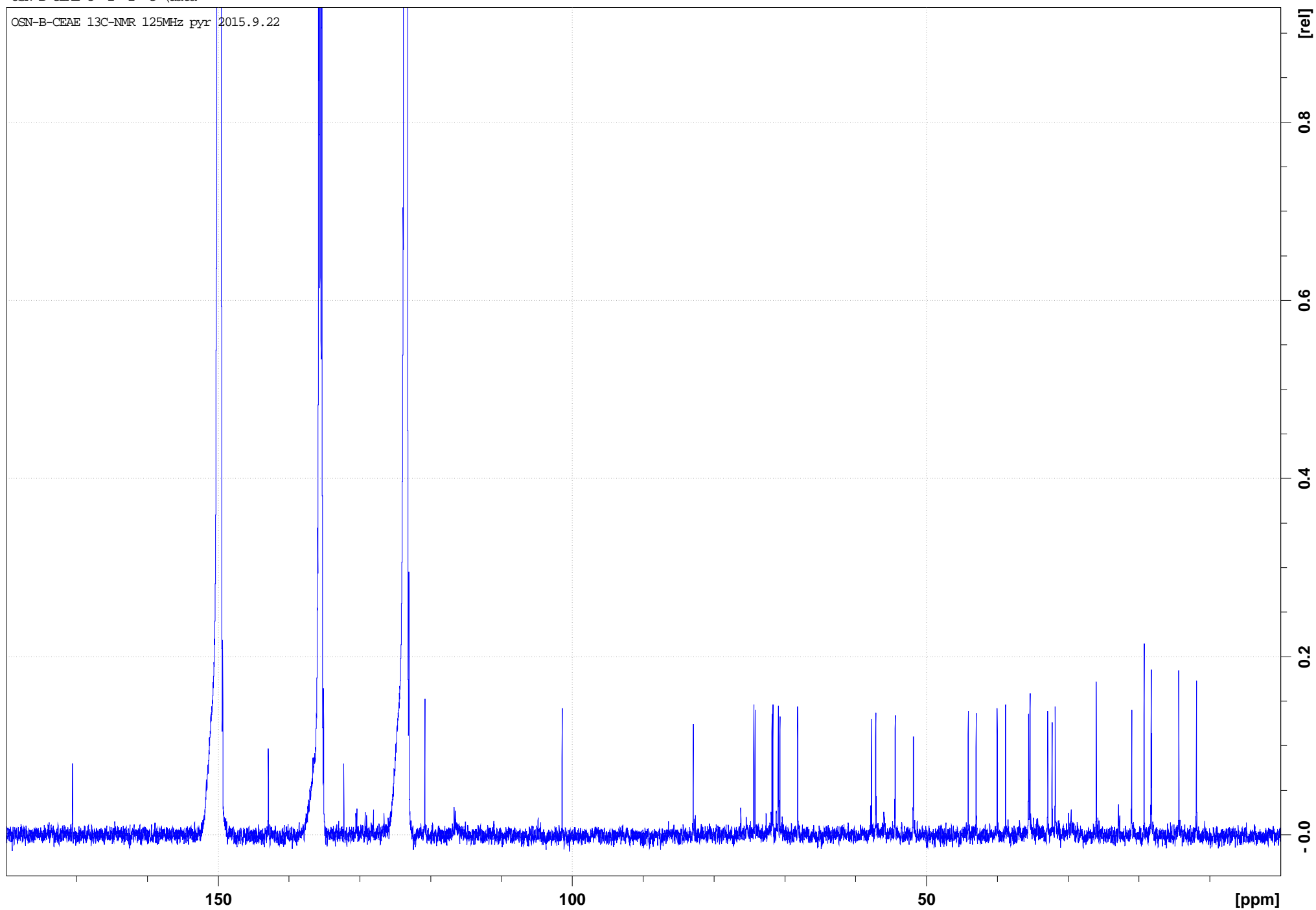

Supplement: Supplementary file 1 [file molecules-22-01243-s001.zip › Compound 5 13C-NMR.pdf]

OSN-B-DFBEI 1 1 C:\data

AV-600 16/2/6

1H OSN-B-DFBEI/C5D5N

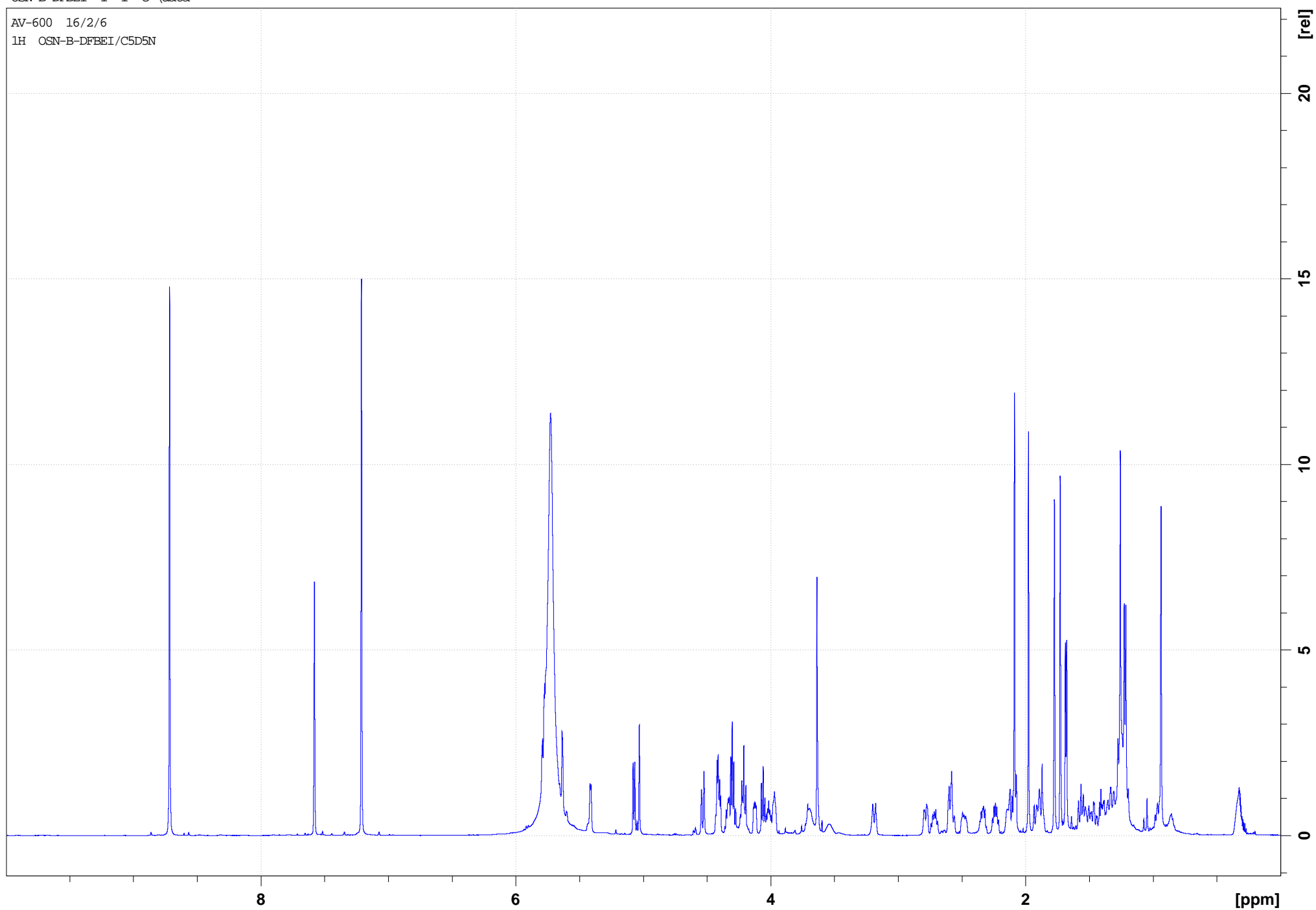

Supplement: Supplementary file 1 [file molecules-22-01243-s001.zip › Compound 6 1H-NMR.pdf]

OSN-B-DFBEI 2 1 C:\data

AV-600 16/2/6

<sup>13</sup>C OSN-B-DFBEI/C5D5N

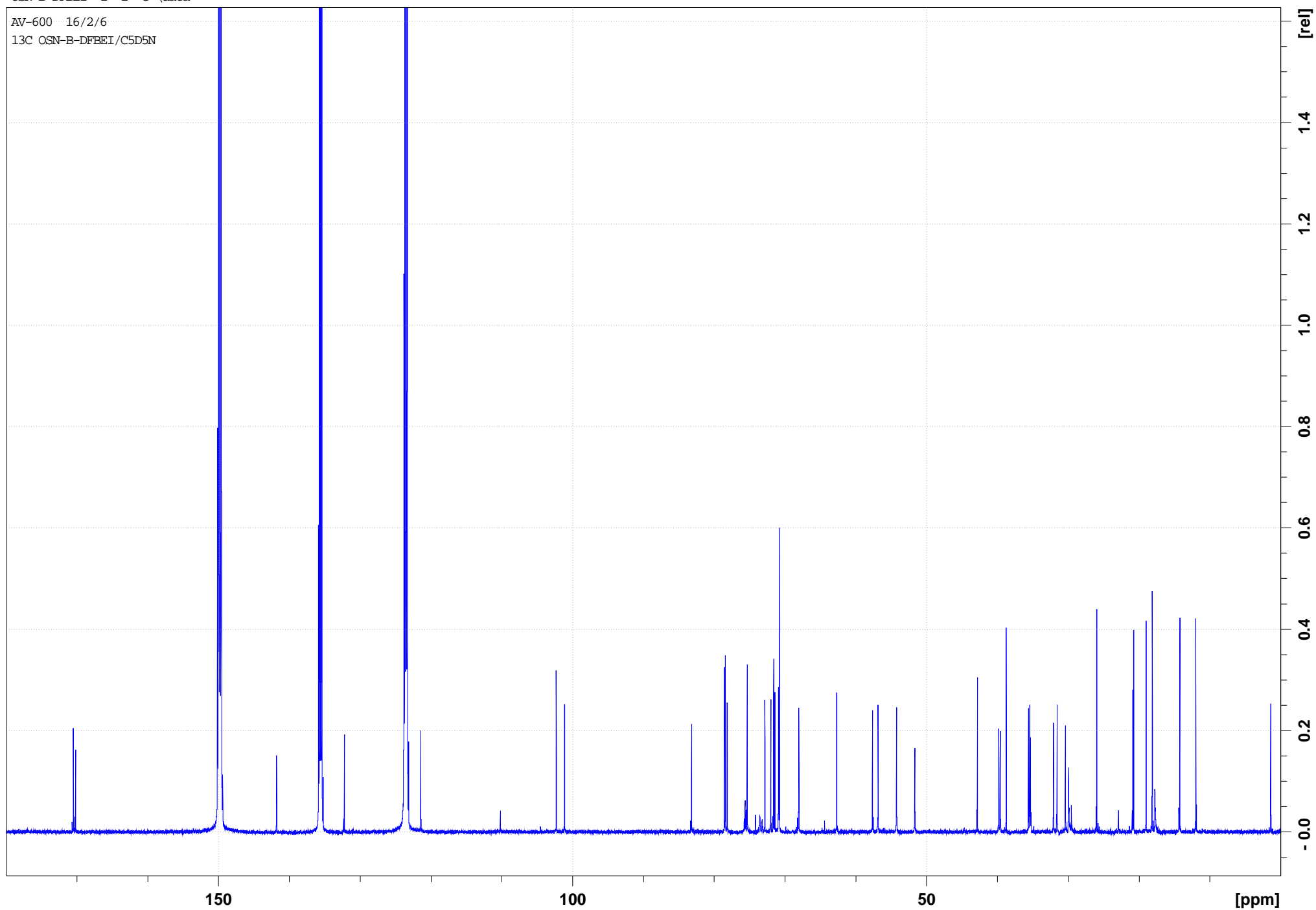

Supplement: Supplementary file 1 [file molecules-22-01243-s001.zip › Compound 6 13C-NMR.pdf]

OSN-B-DFBEIE 1 1 C:\data

AV500 16/8/2

1H OSN-B-DFBEIE/C5D5N

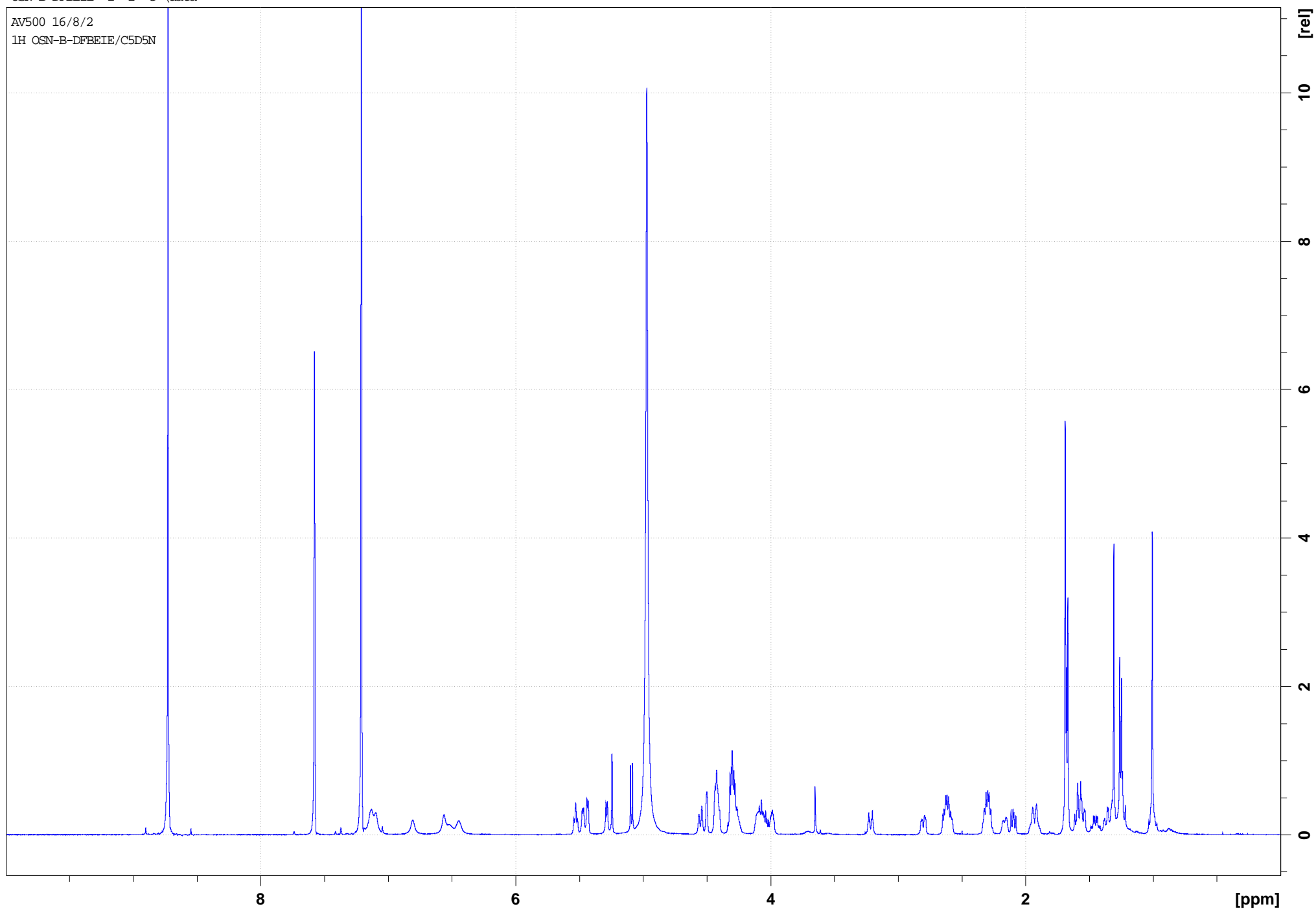

Supplement: Supplementary file 1 [file molecules-22-01243-s001.zip › Compound 6a 1H-NMR.pdf]

OSN-B-DFBEIE 2 1 C:\data

AV500 16/8/2

<sup>13</sup>C OSN-B-DFBEIE/C5D5N

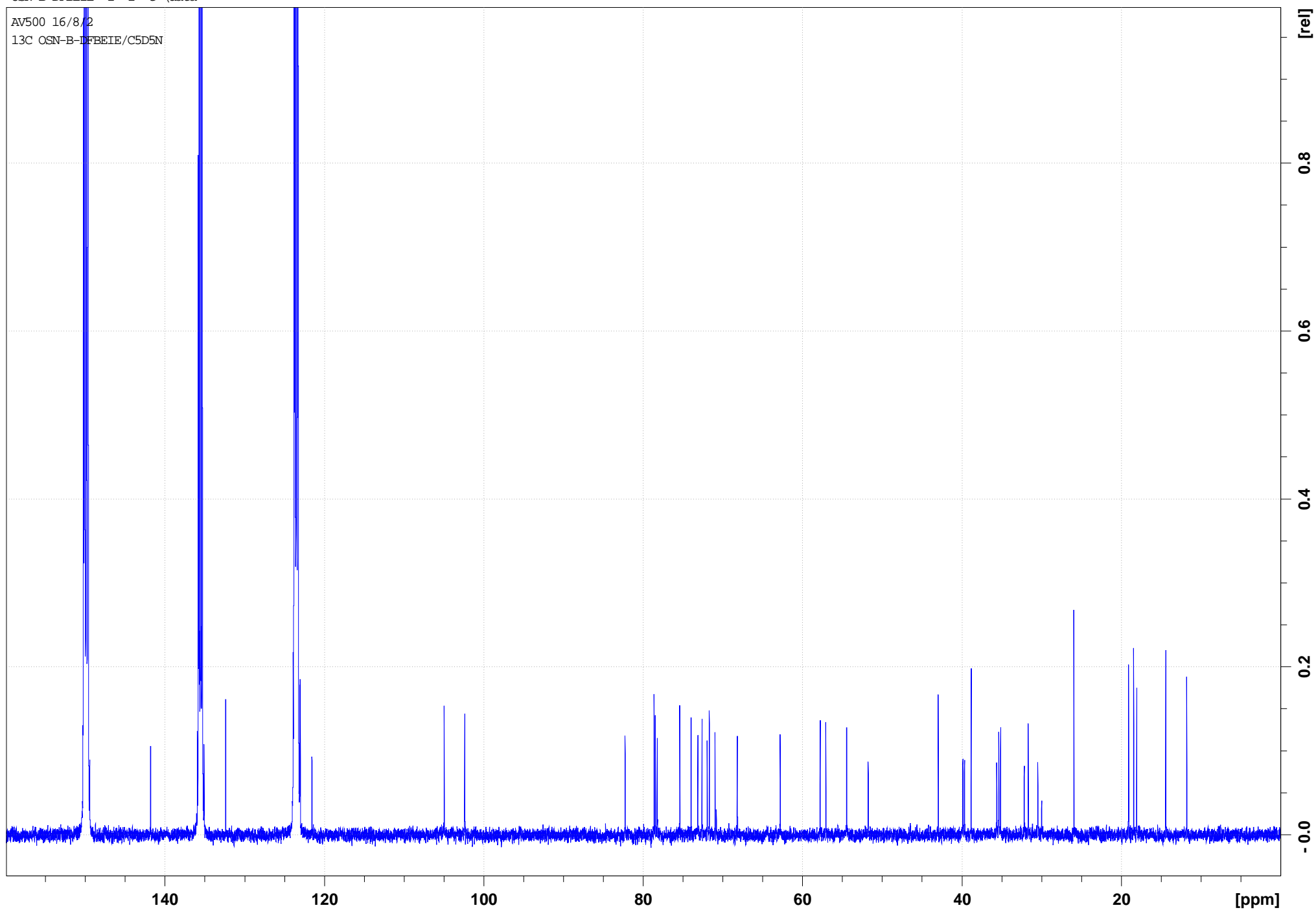

Supplement: Supplementary file 1 [file molecules-22-01243-s001.zip › Compound 6a 13C-NMR.pdf]

OSN-B-DIHBCF 1 1 C:\data

AV-600 15/12/3

1H OSN-B-DIHBCF/C5D5N

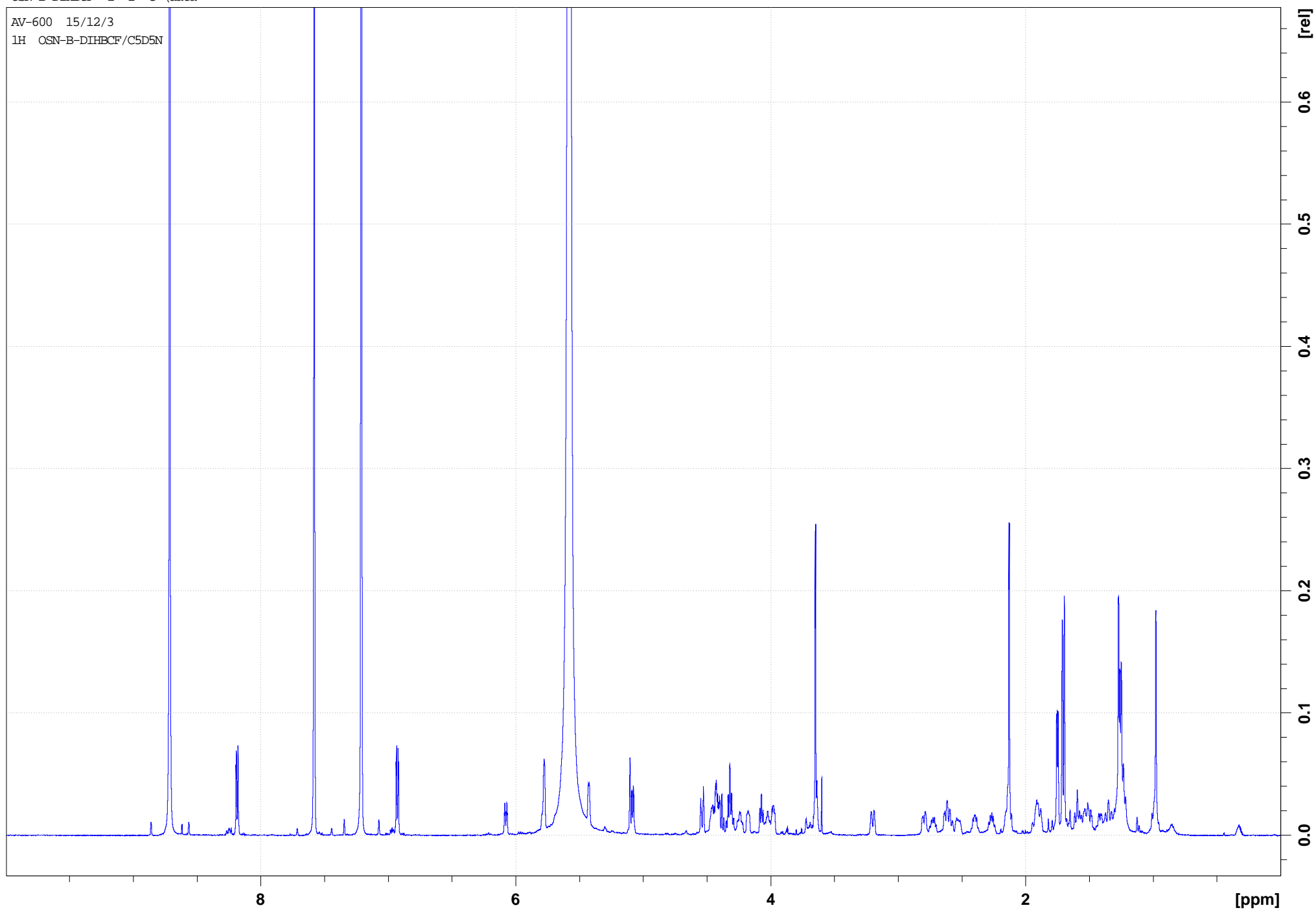

Supplement: Supplementary file 1 [file molecules-22-01243-s001.zip › Compound 7 1H-NMR.pdf]

OSN-B-DIHBCF 2 1 C:\data

AV-600 15/12/3

<sup>13</sup>C OSN-B-DIHBCF/C<sub>5</sub>D<sub>5</sub>N

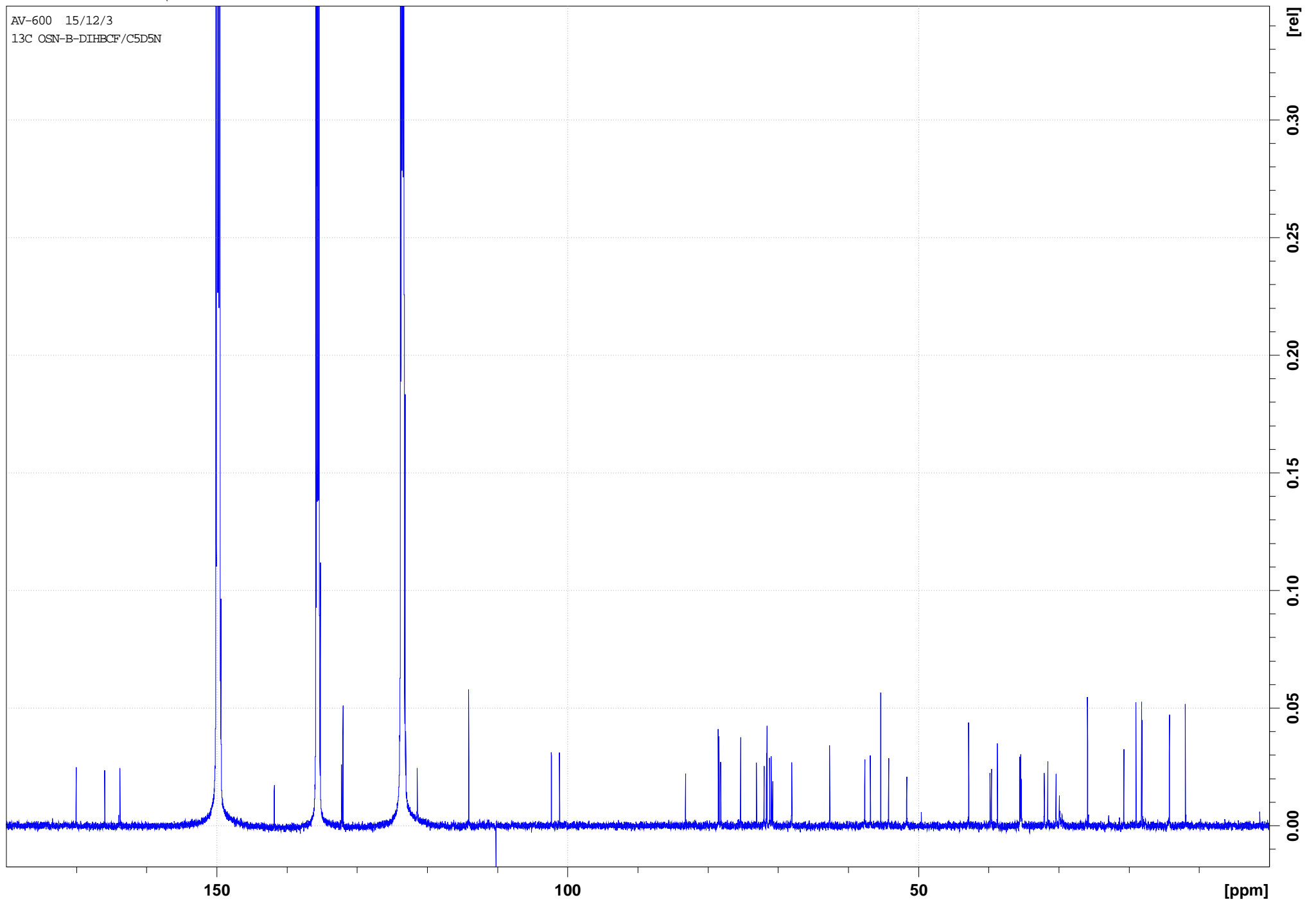

Supplement: Supplementary file 1 [file molecules-22-01243-s001.zip › Compound 7 13C-NMR.pdf]

OSN-B-DIGI 1 1 C:\data

AV-600 16/2/17

1H OSN-B-DIGI/C5D5N

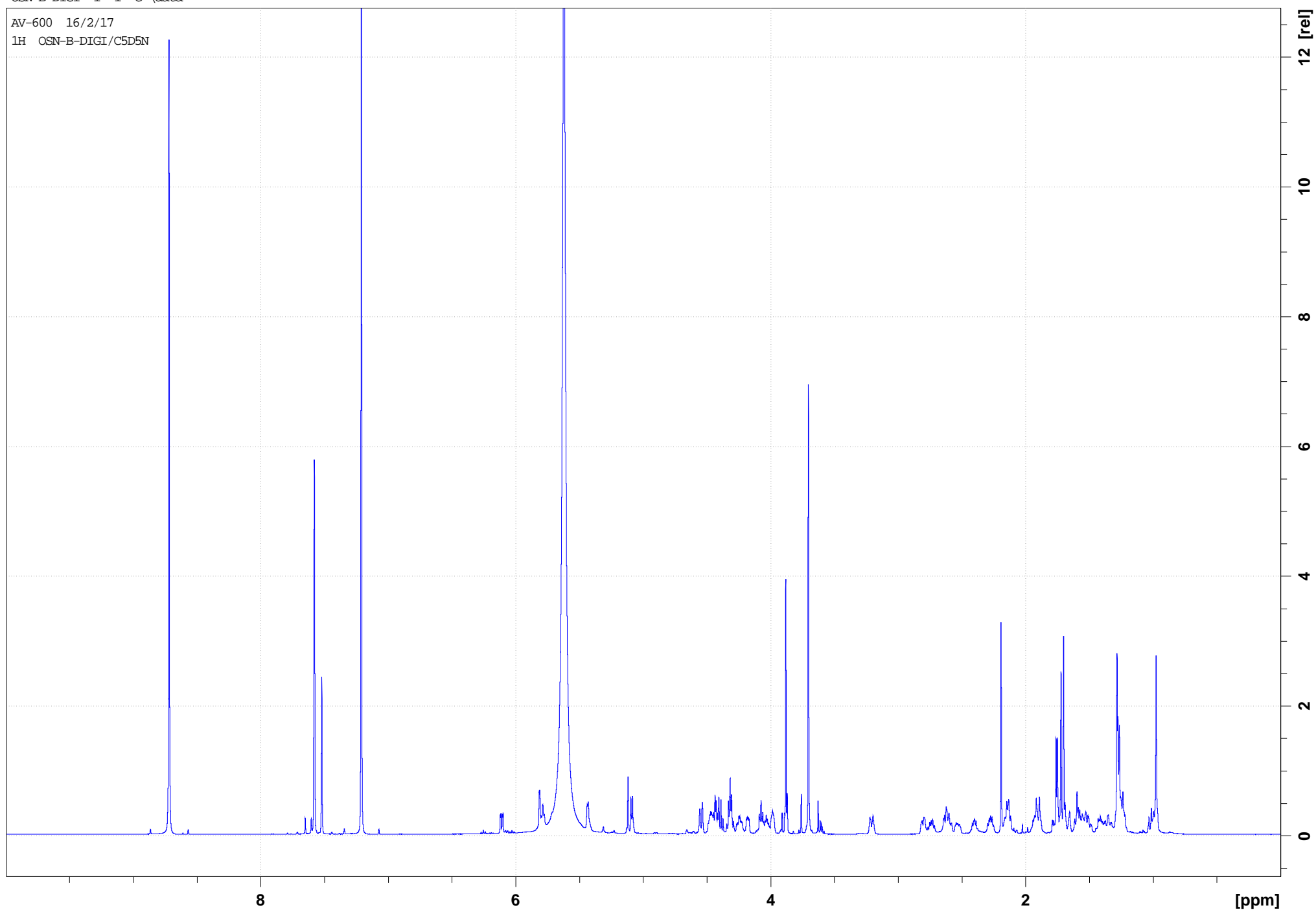

Supplement: Supplementary file 1 [file molecules-22-01243-s001.zip › Compound 8 1H-NMR.pdf]

OSN-B-DIGI 2 1 C:\data

AV-600 16/2/18

<sup>13</sup>C OSN-B-DIGI/C5D5N

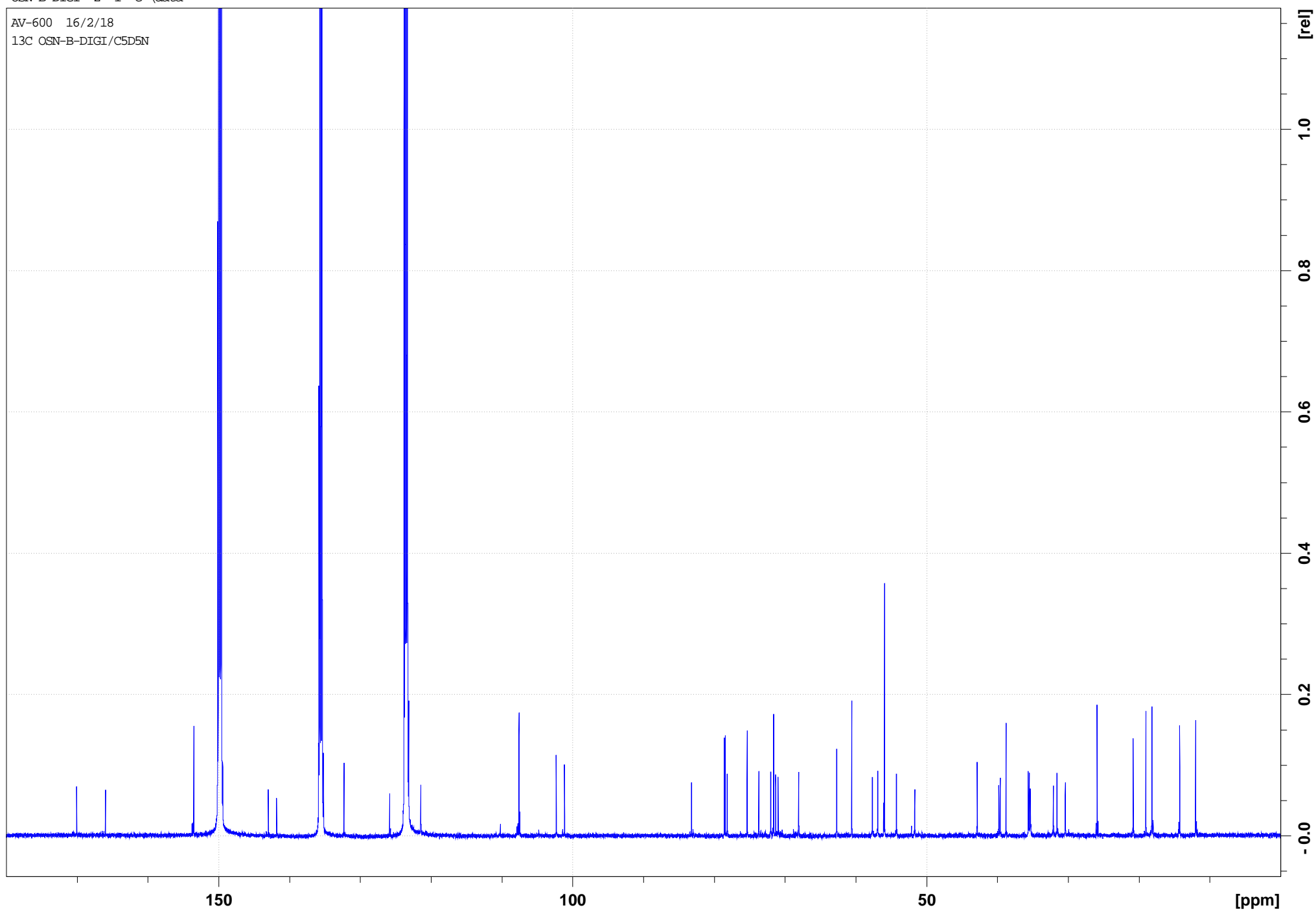

Supplement: Supplementary file 1 [file molecules-22-01243-s001.zip › Compound 8 13C-NMR.pdf]

OSN-B-FDG-c 1 1 C:\data

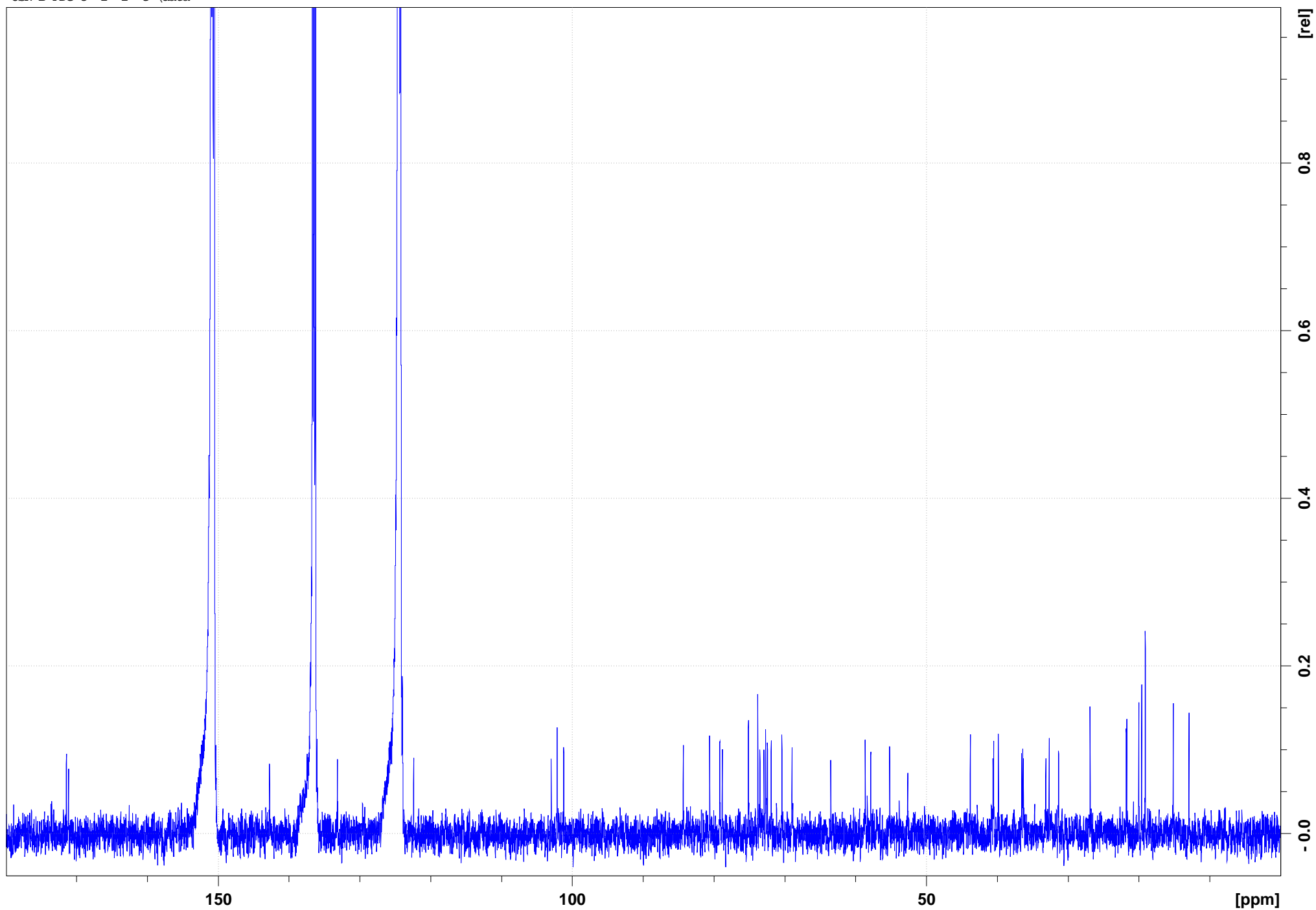

Supplement: Supplementary file 1 [file molecules-22-01243-s001.zip › Compound 9 13C-NMR.pdf]

OSN-B-DINP-h 1 1 C:\data

OSN-B-DINP 1H-NMR C5D5N 500MHz 2014/04/15

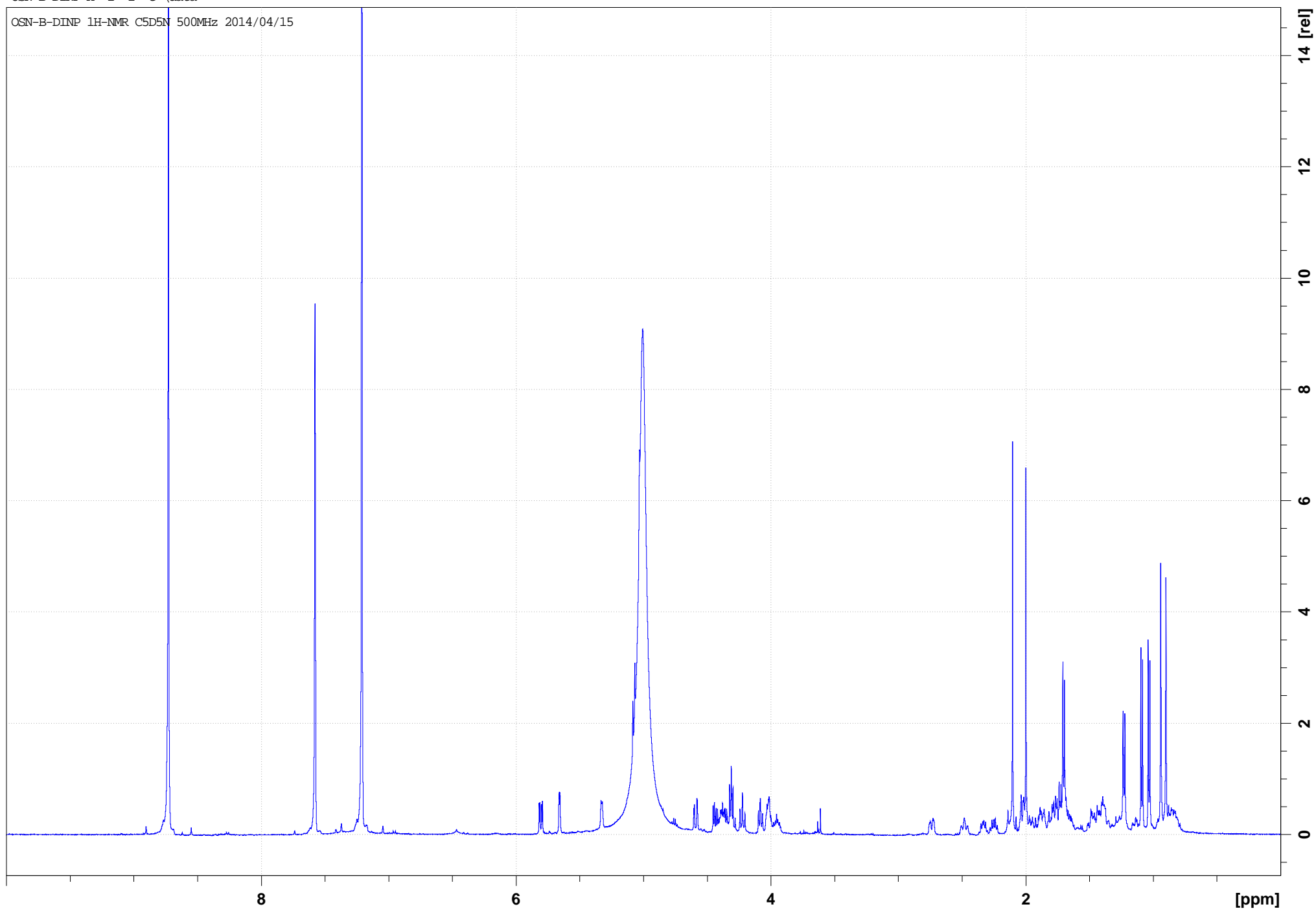

Supplement: Supplementary file 1 [file molecules-22-01243-s001.zip › Compound 10 1H-NMR.pdf]

OSN-B-DINP-c 1 1 C:\data  
OSN-B-DINP 13C-NMR C5D5N 2015.04.15

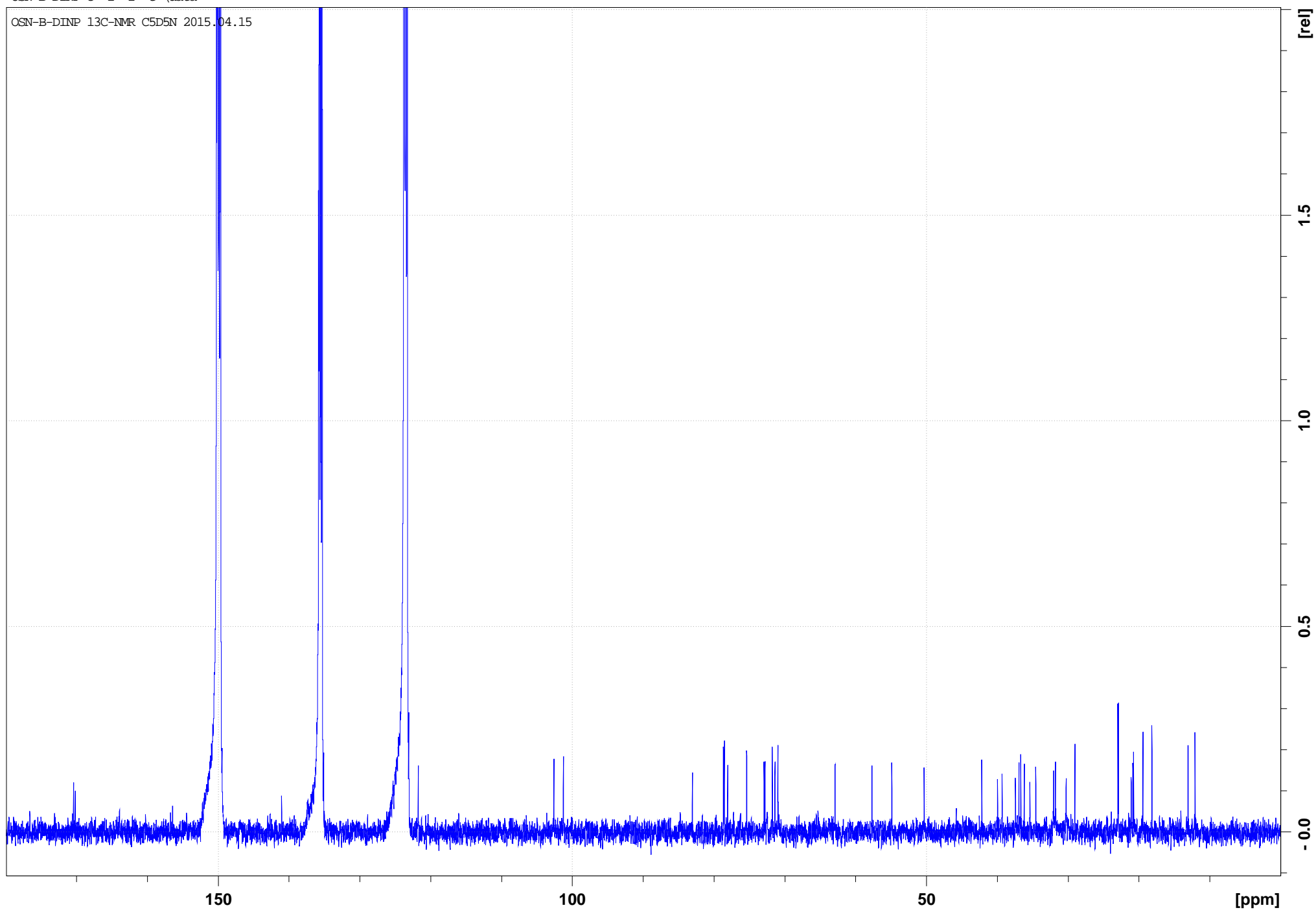

Supplement: Supplementary file 1 [file molecules-22-01243-s001.zip › Compound 10 13C-NMR.pdf]

OSN-B-DIOCB 1 1 C:\data

AV-600 16/1/27

1H OSN-B-DIOCB/C5D5N

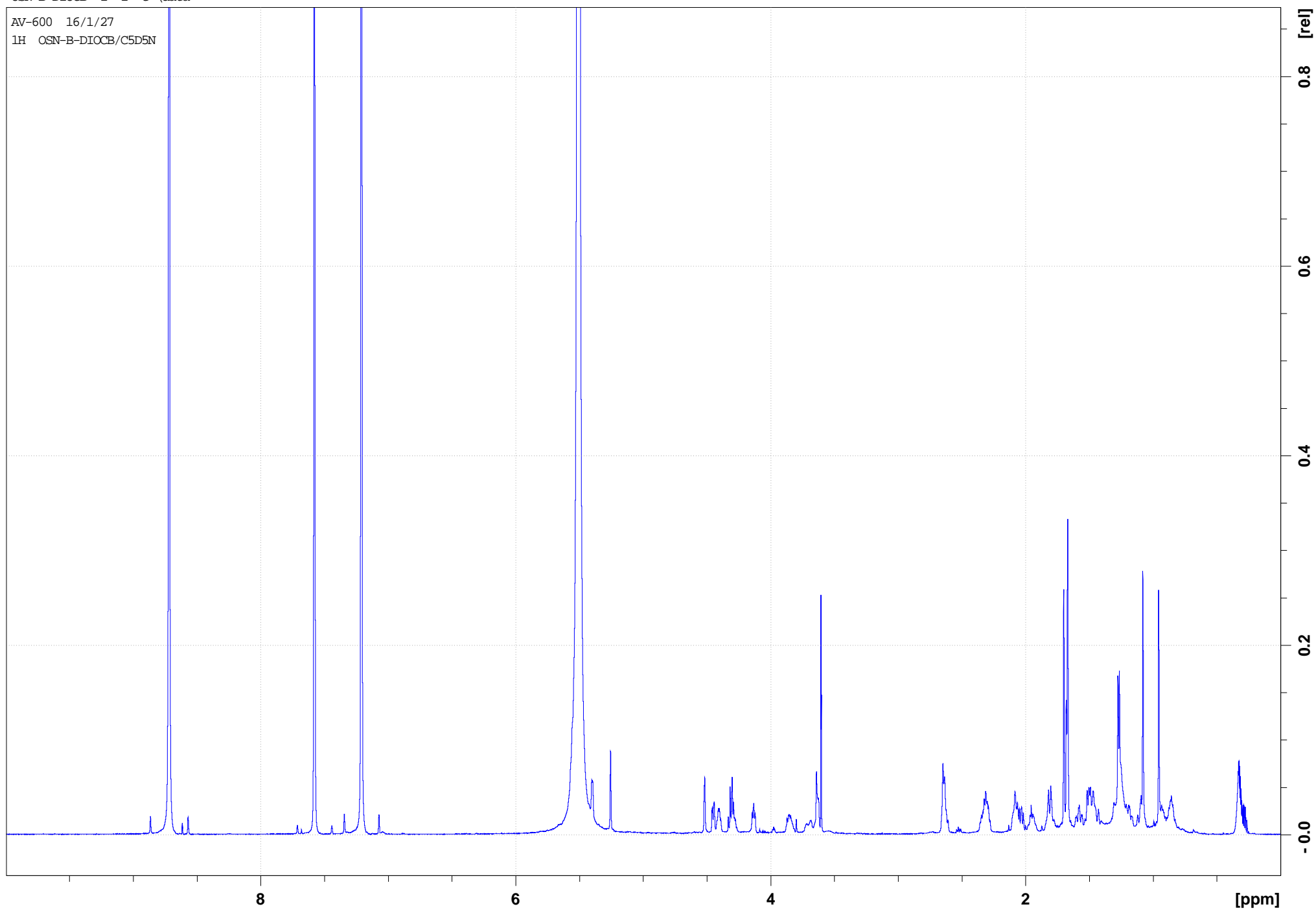

Supplement: Supplementary file 1 [file molecules-22-01243-s001.zip › Compound 11 1H-NMR.pdf]

OSN-B-DIOCB 2 1 C:\data

AV-600 16/1/28

<sup>13</sup>C OSN-B-DIOCB/C5D5N

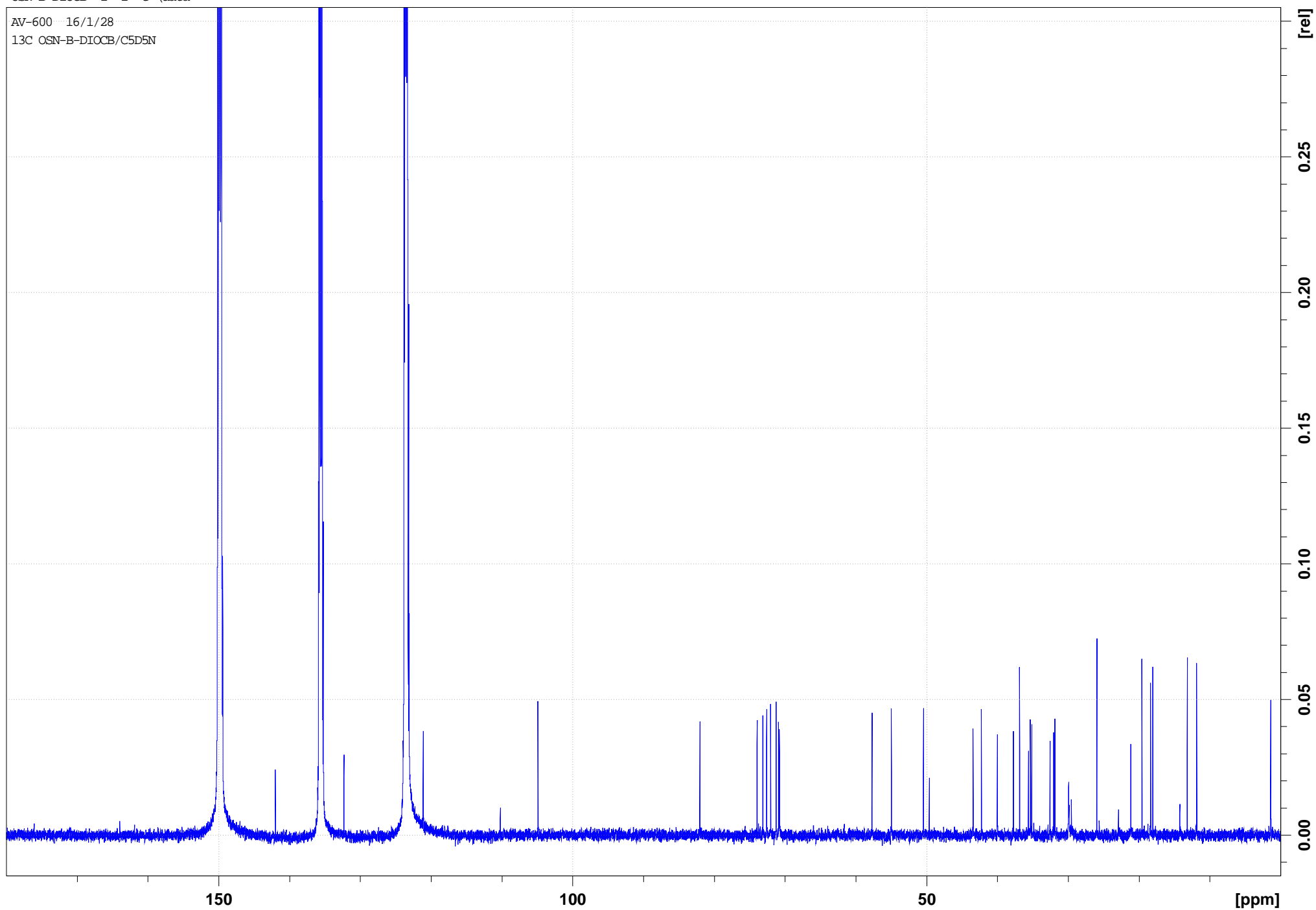

Supplement: Supplementary file 1 [file molecules-22-01243-s001.zip › Compound 11 13C-NMR.pdf]

OSN-B-DILEBB 1 1 C:\data

AV-600 15/10/9

1H OSN-B-DILEBB/C5D5N

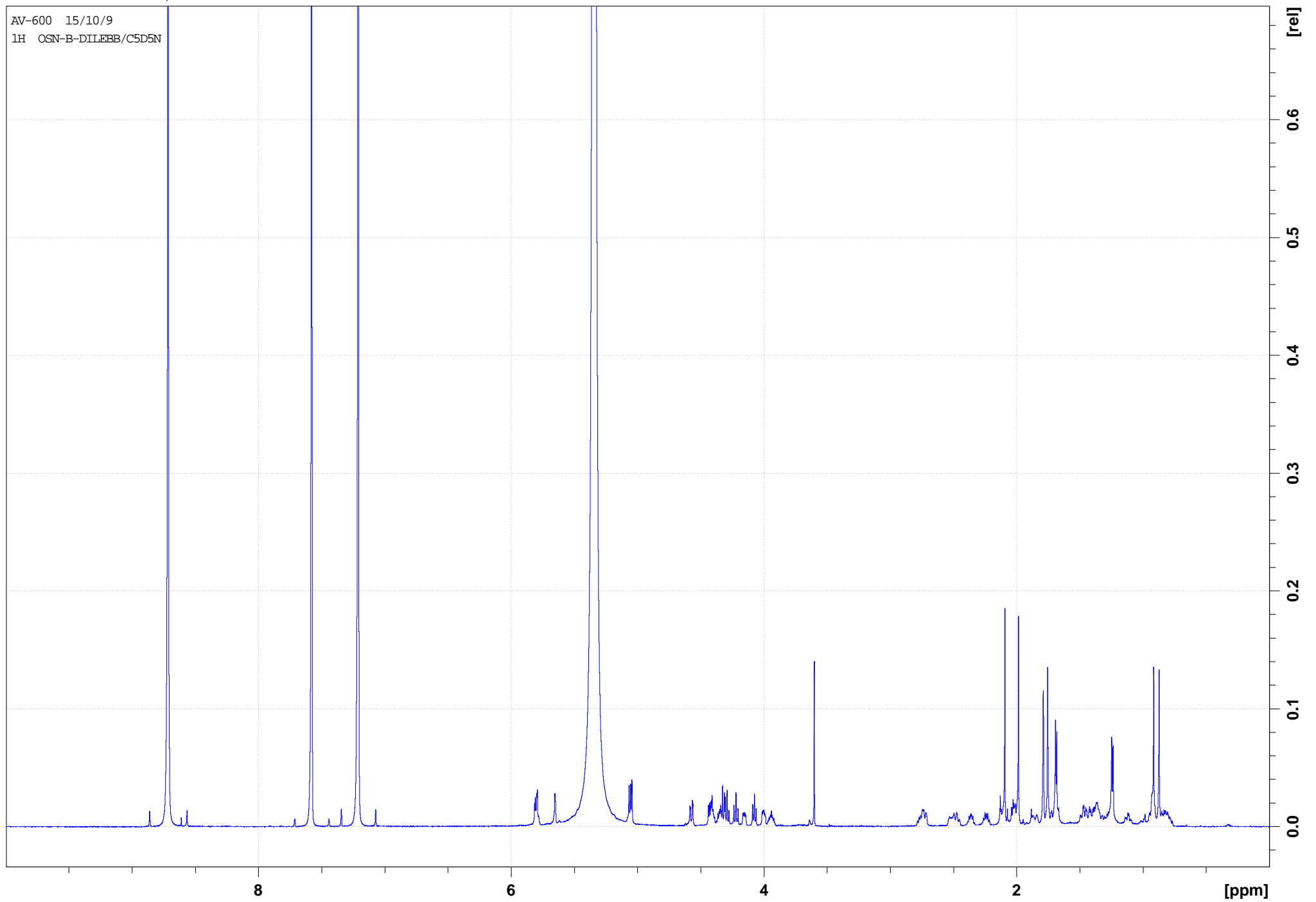

Supplement: Supplementary file 1 [file molecules-22-01243-s001.zip › Compound 12 1H-NMR.pdf]

OSN-B-DILEBB 2 1 C:\data

AV-600 15/10/9

<sup>13</sup>C OSN-B-DILEBB/C<sub>5</sub>D<sub>5</sub>N

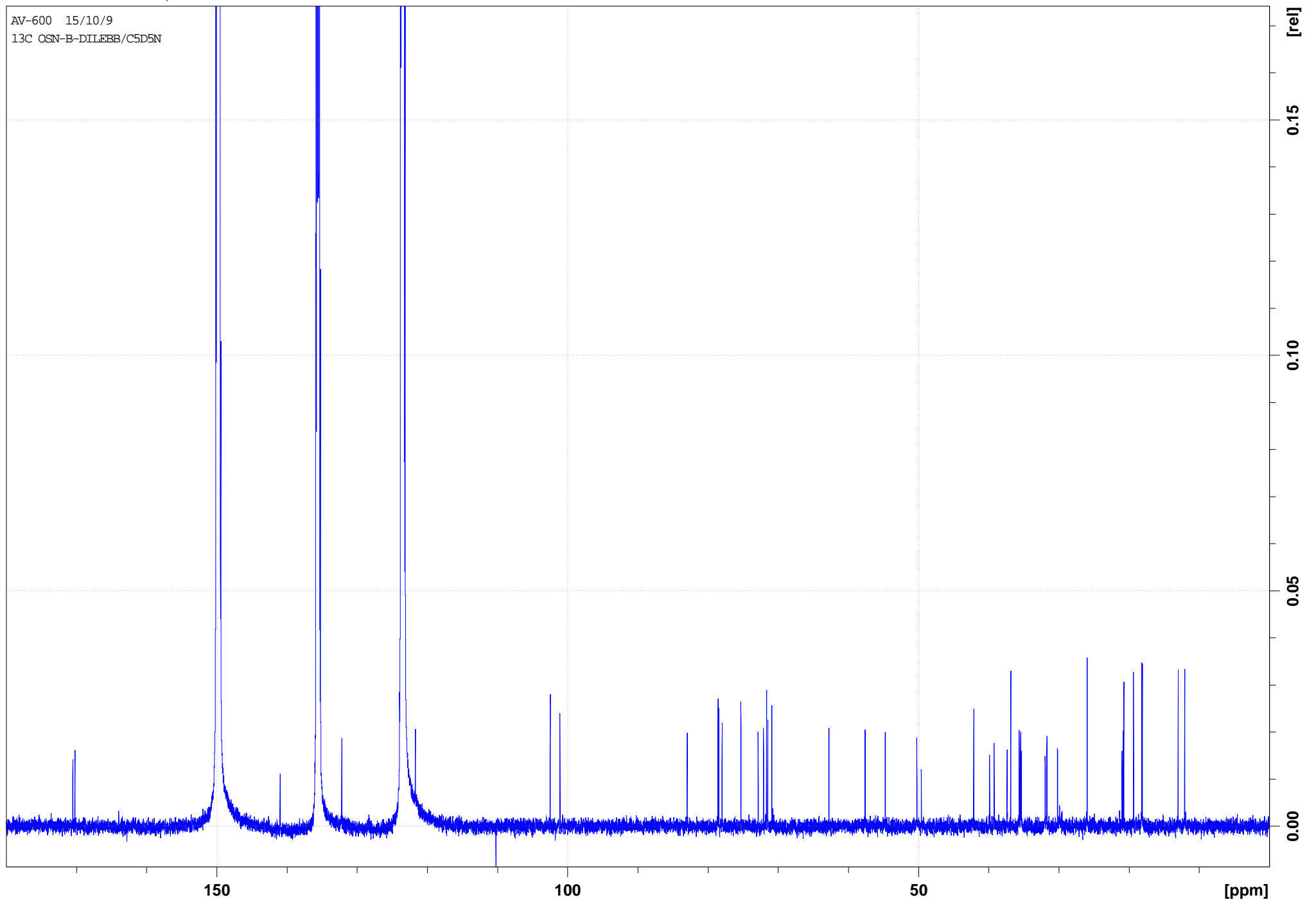

Supplement: Supplementary file 1 [file molecules-22-01243-s001.zip › Compound 12 13C-NMR.pdf]

OSN-B-FDCC-h 1 1 C:\data  
OSN-B-FDCC 1H-NMR 500MHz C5D5N 2014.1.22

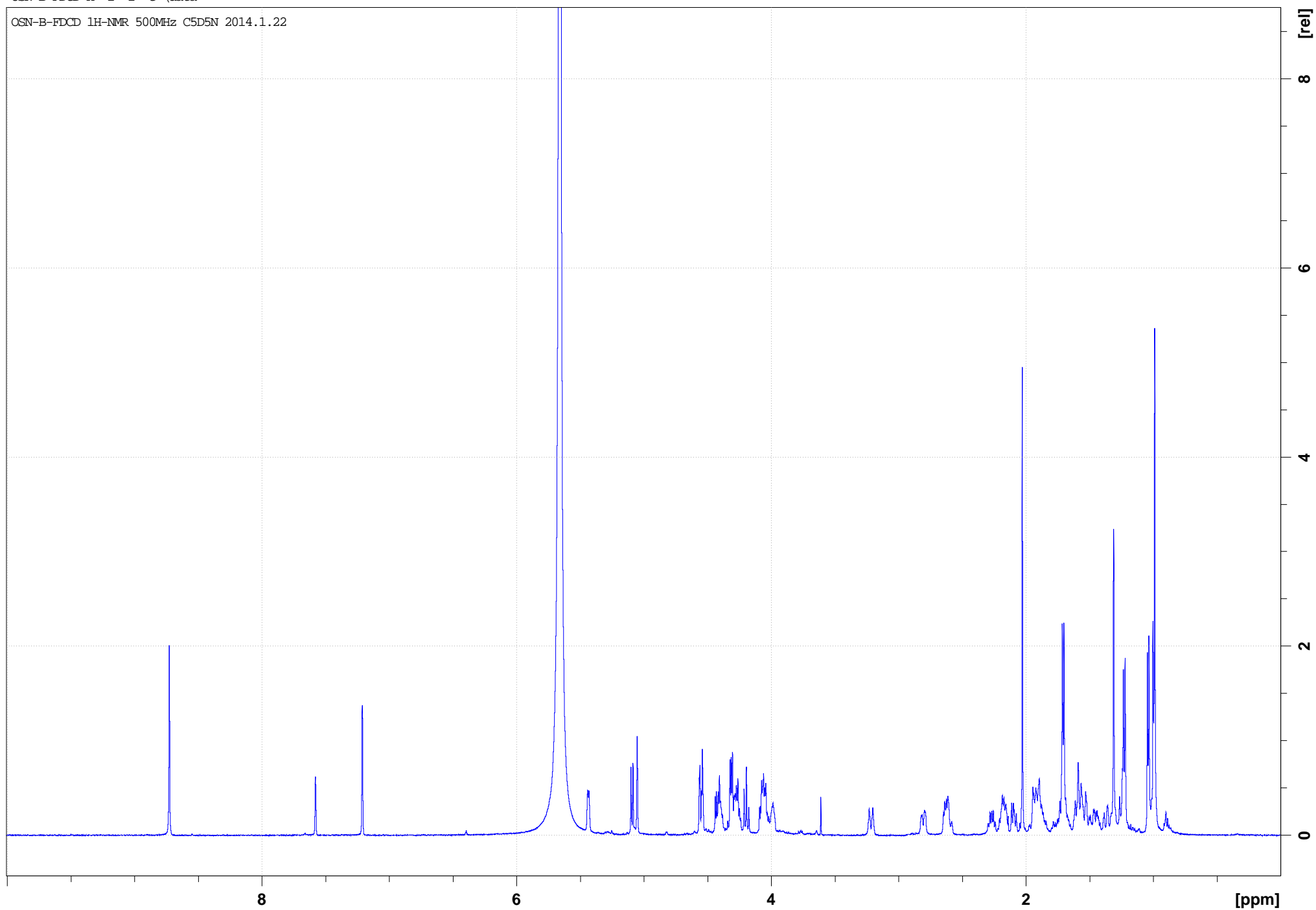

Supplement: Supplementary file 1 [file molecules-22-01243-s001.zip › Compound 1 1H-NMR.pdf]

OSN-B-FDCC-c 1 1 C:\data  
OSN-B-FDCC 13C-NMR C5D5N 500MHz 2014.2.5

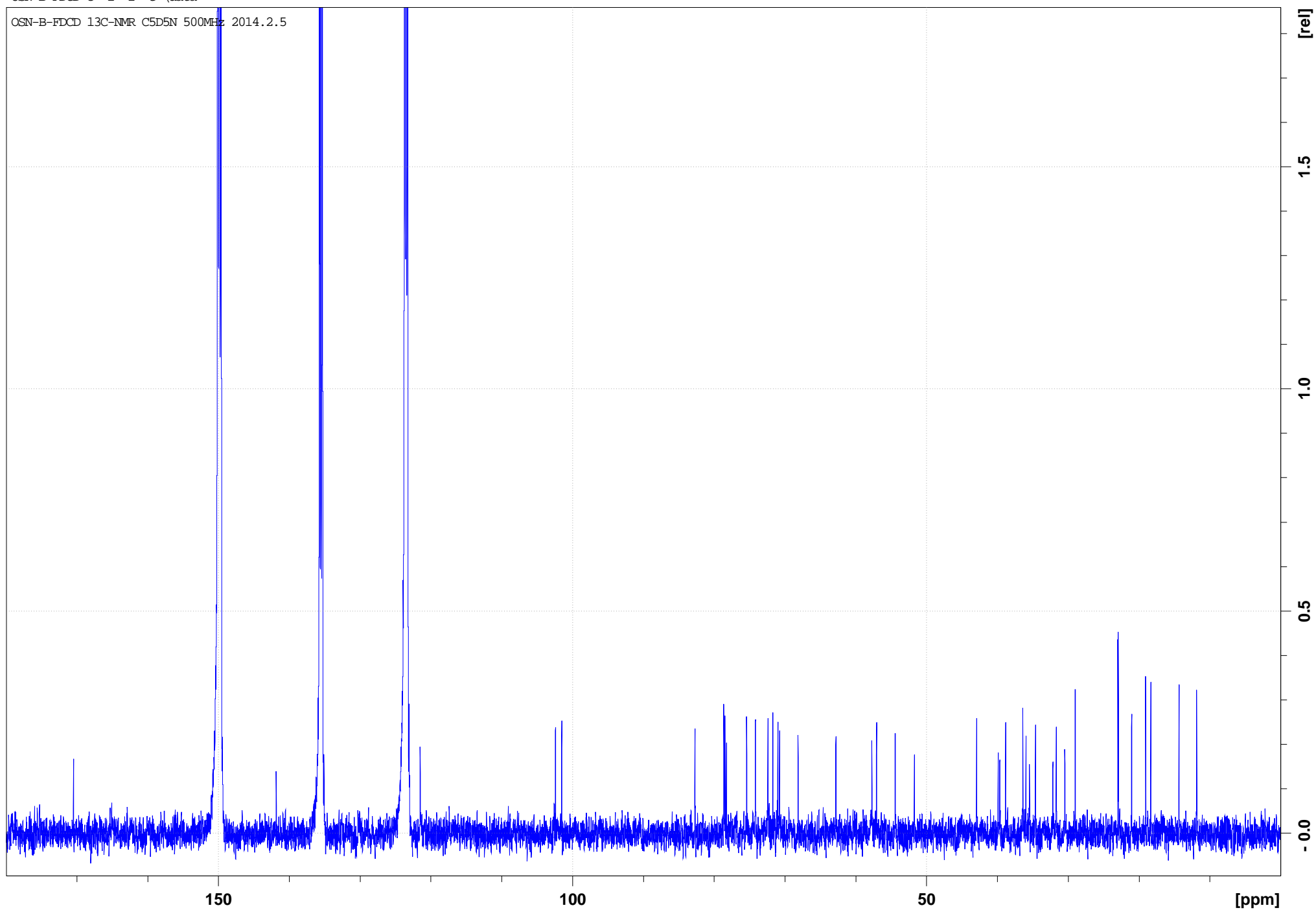

Supplement: Supplementary file 1 [file molecules-22-01243-s001.zip › Compound 1 13C-NMR.pdf]

OSN-B-FDCC-b-h 1 1 C:\data

OSN-B-FDCC-b 1H-NMR 500MHz 2014.05.17 C5D5N

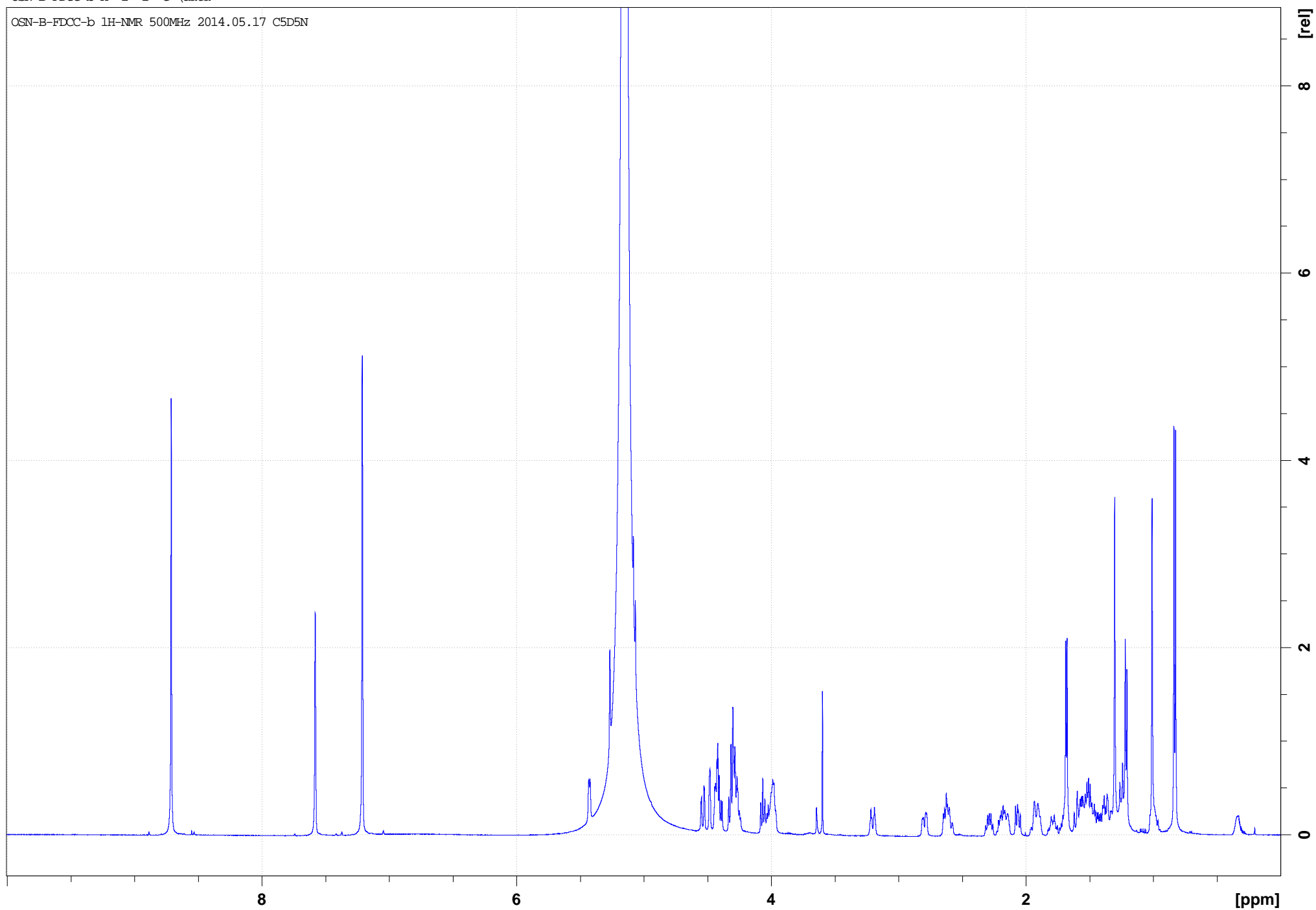

Supplement: Supplementary file 1 [file molecules-22-01243-s001.zip › Compound 1a 1H-NMR.pdf]

OSN-B-FDCC-b-c 1 1 C:\data

OSN-B-FDCC-b C5D5N 500MHz 2014.05.17 13C-NMR

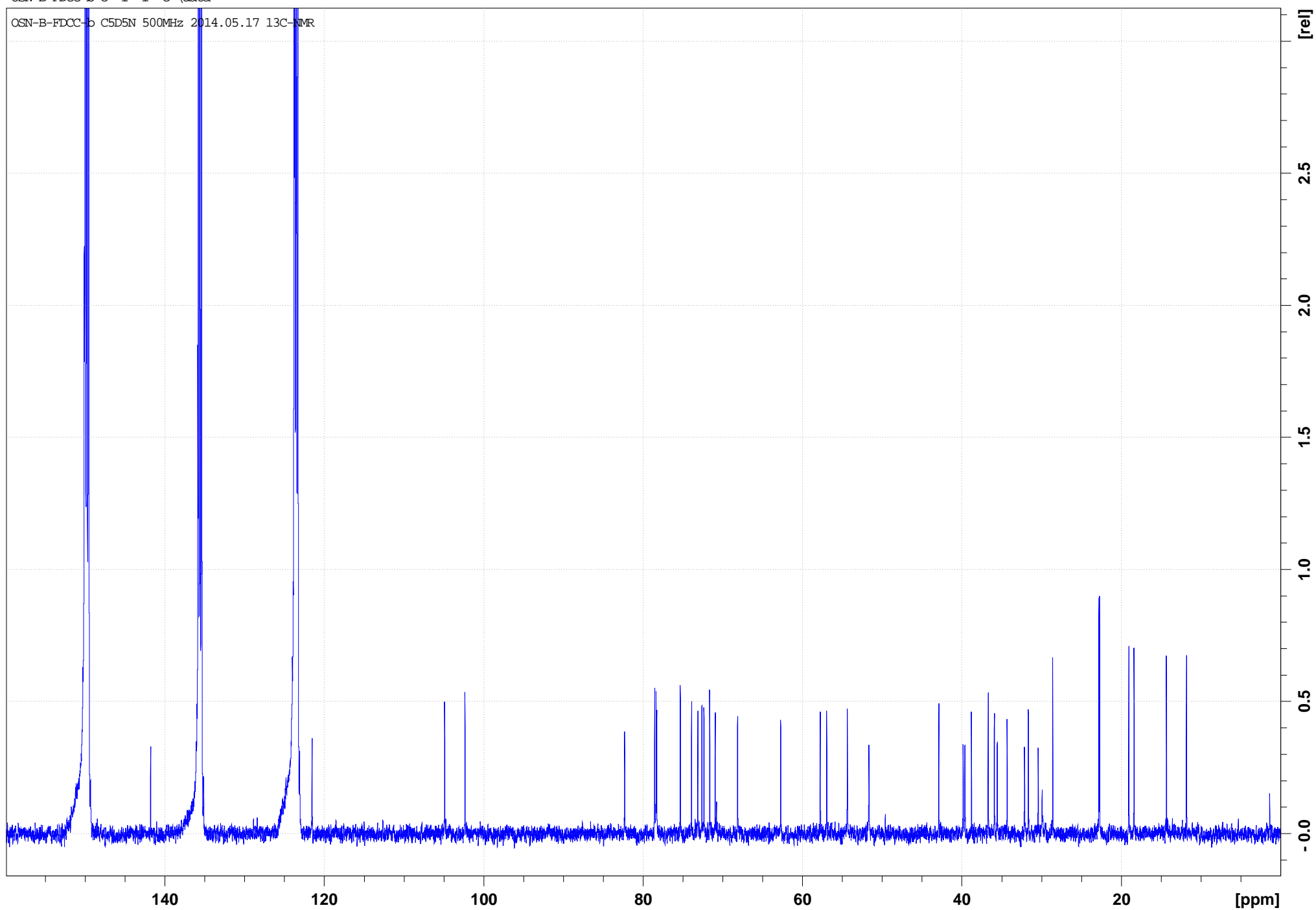

Supplement: Supplementary file 1 [file molecules-22-01243-s001.zip › Compound 1a 13C-NMR.pdf]

OSN-B-DIDB 1 1 C:\data

AV-600 15/8/23

1H OSN-B-DIDB/C5D5N

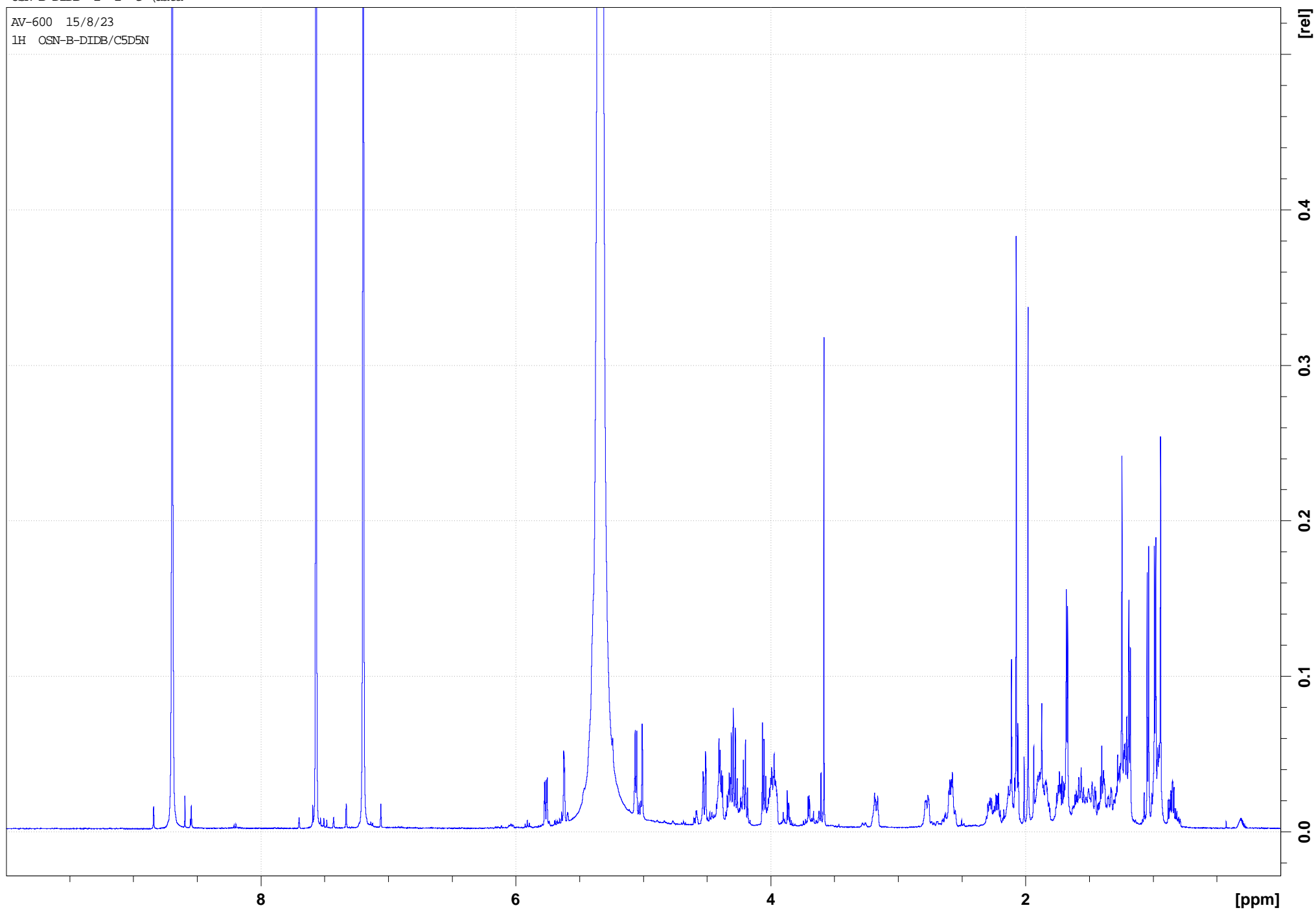

Supplement: Supplementary file 1 [file molecules-22-01243-s001.zip › Compound 2 1H-NMR.pdf]

OSN-B-DIDB 2 1 C:\data

AV-600 15/8/24

<sup>13</sup>C OSN-B-DIDB/C5D5N

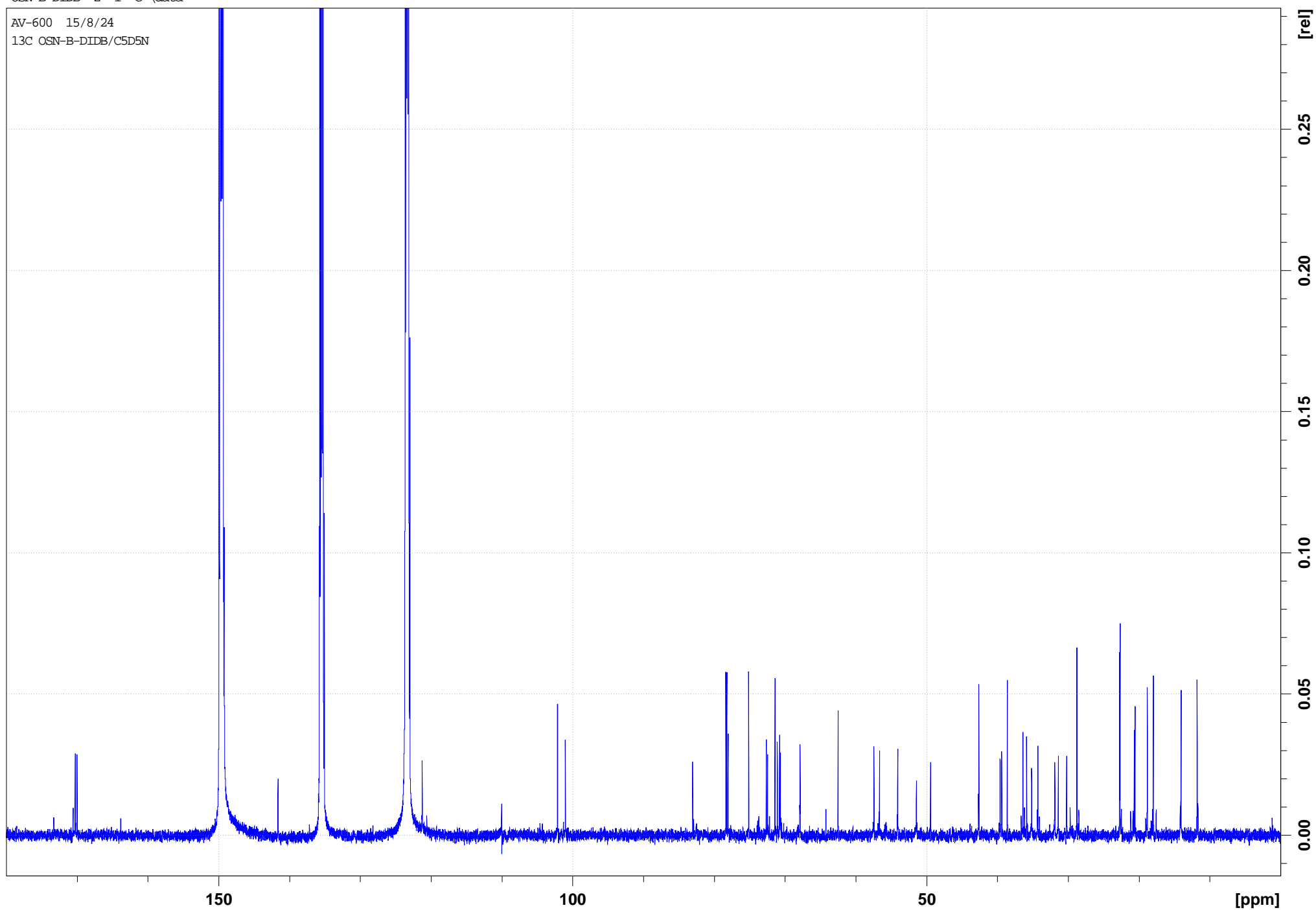

Supplement: Supplementary file 1 [file molecules-22-01243-s001.zip › Compound 2 13C-NMR.pdf]
